# Supplementary material for: Identification of novel driver risk genes in CNV loci associated with neurodevelopmental disorders
Source: HGG Adv. 2024 Jun 6;5(3):100316. doi: 10.1016/j.xhgg.2024.100316 (PMC11264174; doi:10.1016/j.xhgg.2024.100316)
Supplement: Document S1. Figures S1–S4, supplemental methods, and supplemental information [file mmc1.pdf]

HGGA, Volume *5*

Supplemental information

Identification of novel driver risk genes in CNV loci associated with neurodevelopmental disorders

Sara Azidane, Xavier Gallego, Lynn Durham, Mario Cáceres, Emre Guney, and Laura Pérez-Cano

Supplemental information:

Table of Contents

Supplemental figures: 2

Supplemental Figure 1. Density plot showing the distribution of normalized RNA-seq counts across the different developmental stages. 2

Supplemental Figure 2. Number of highly expressed genes in different brain regions across all human development. 3

Supplemental Figure 3. 36 qDSGs are highly expressed in key areas of the brain across all developmental stages. 4

Supplemental Figure 4. Bar plot depicting Odds Ratios from significantly highly expressed qDSGs in key areas of the brain across all developmental stages. 5

Supplemental Methods: 6

Meta-analysis methodology 6

Standardization procedure 6

Patient Identifiers standardization 7

Supplemental Information: 8

Shared etiology of NDDs [*1] 8

Details about other validated NDD-risk genes [*2] 8

Supplemental references: 9

Supplemental figures:

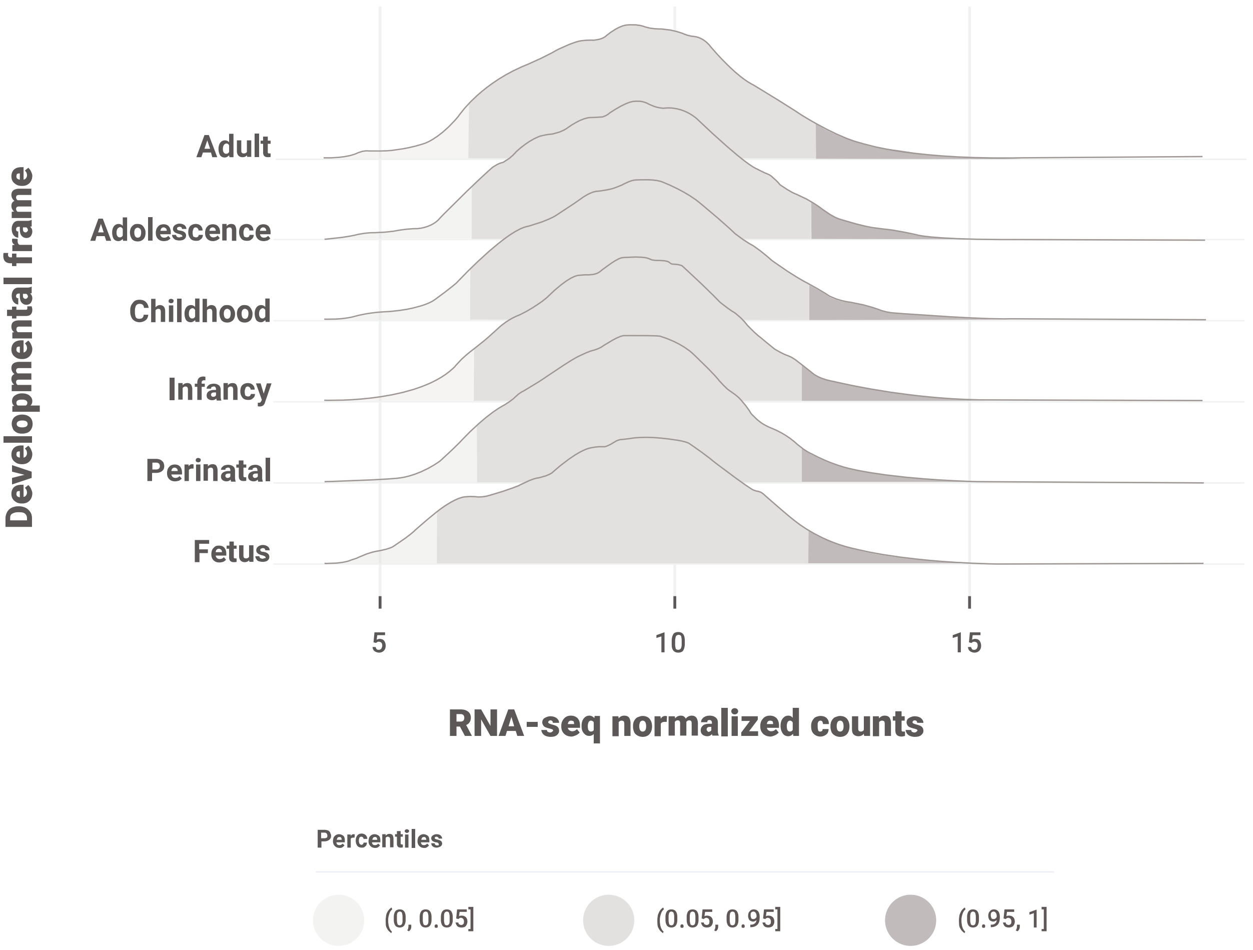

Supplemental Figure 1. Density plot showing the distribution of normalized RNA-seq counts across the different developmental stages.

In different colors are shown the perceptual ranges used to classify high expression (yellow, 95% percentile), medium expression (grey, 5% to 95%) and low expression (blue, 5% percentile) genes.

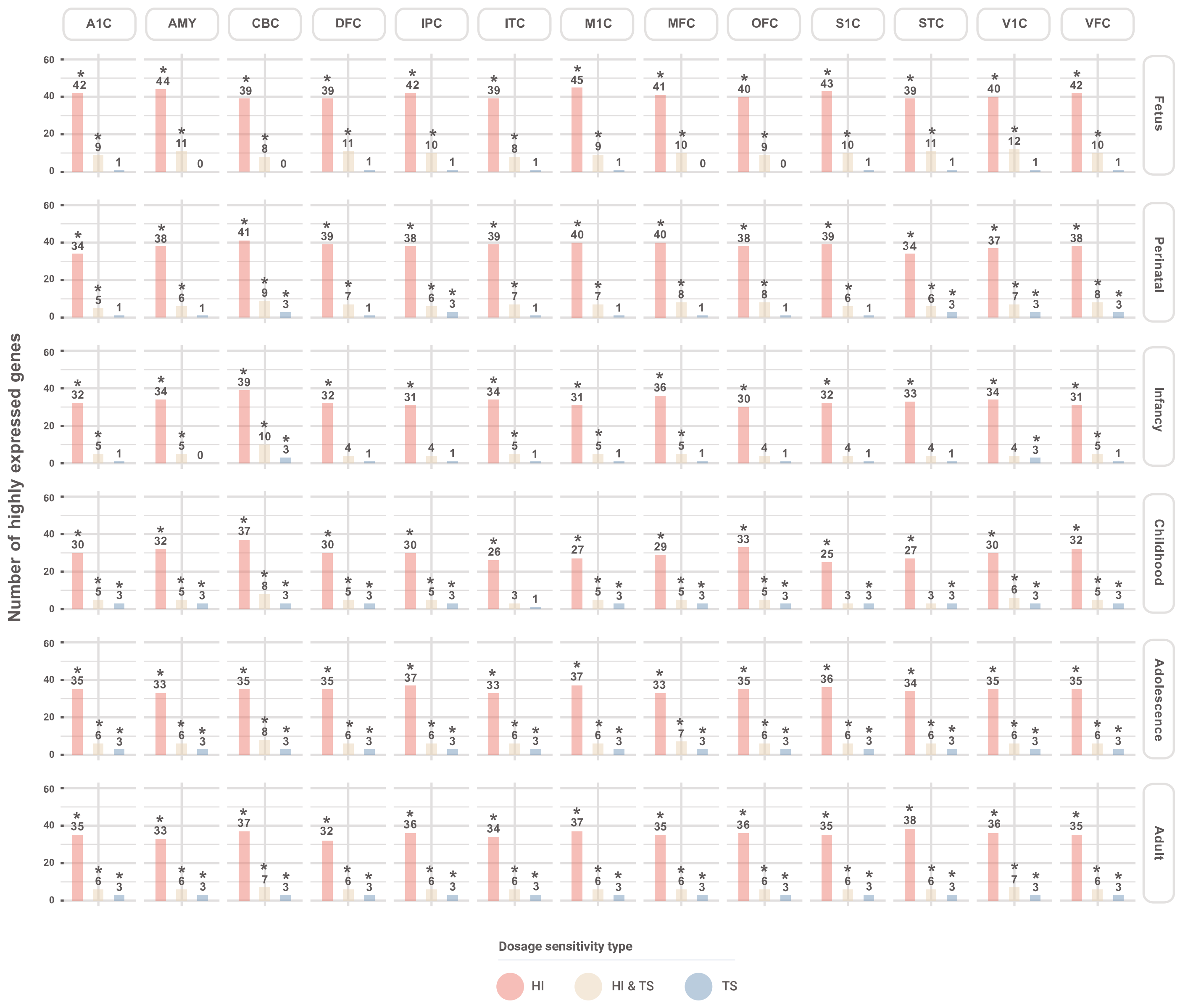

Supplemental Figure 2. Number of highly expressed genes in different brain regions across all human development.

Bar plot showing the number of highly expressed genes, i.e., with a range of expression in the top 5% percentile, in each brain region at different stages of development. The asterisk accompanying the bars indicates that this number of genes is significantly representative, i.e., the most highly expressed genes in that brain tissue at that developmental stage are enriched with our qDSGs.

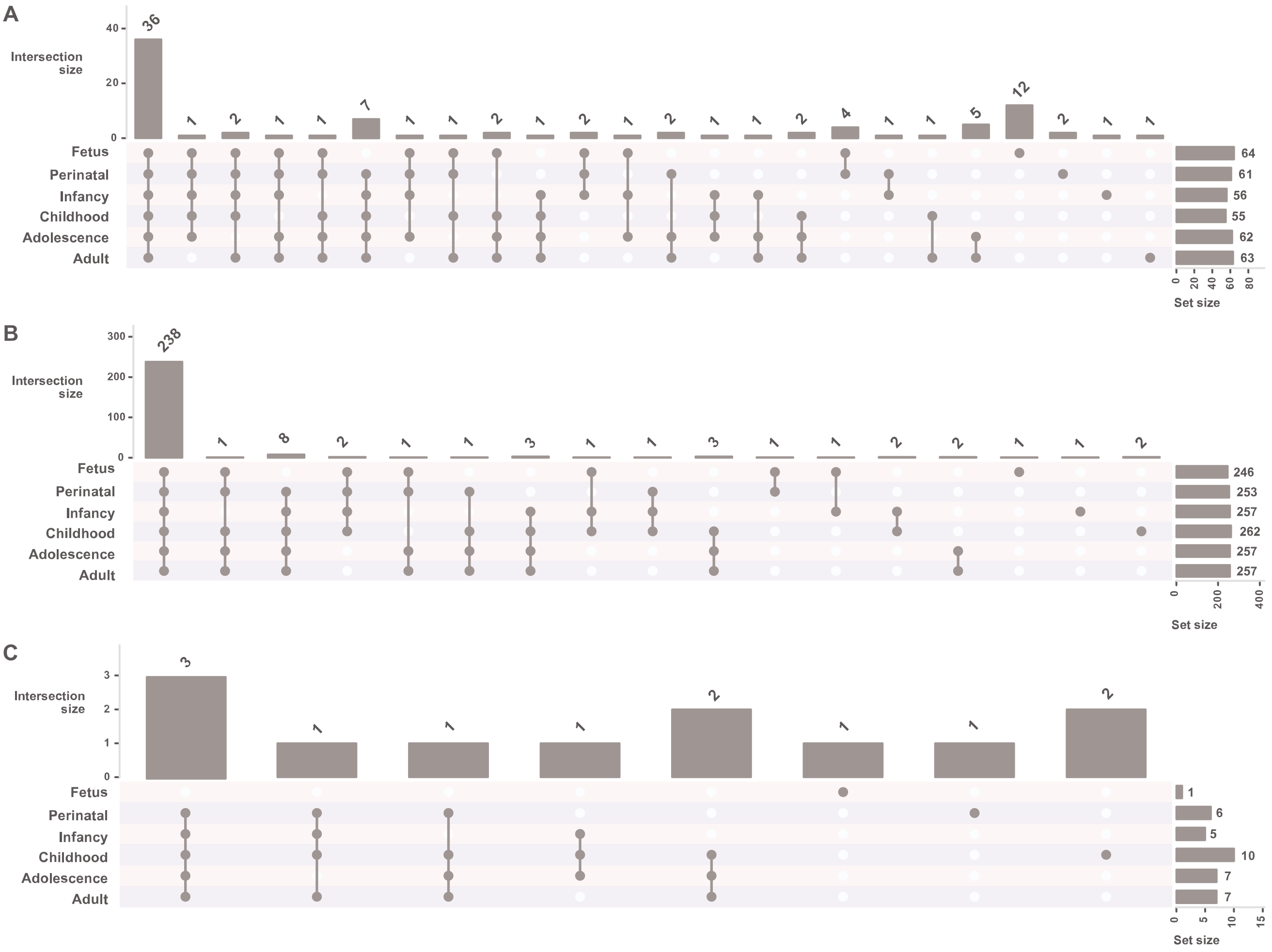

Supplemental Figure 3. 36 qDSGs are highly expressed in key areas of the brain across all developmental stages.

Venn diagram on the number of genes in each expression rank, i.e. high (A), medium (B) or low (C) expressed.

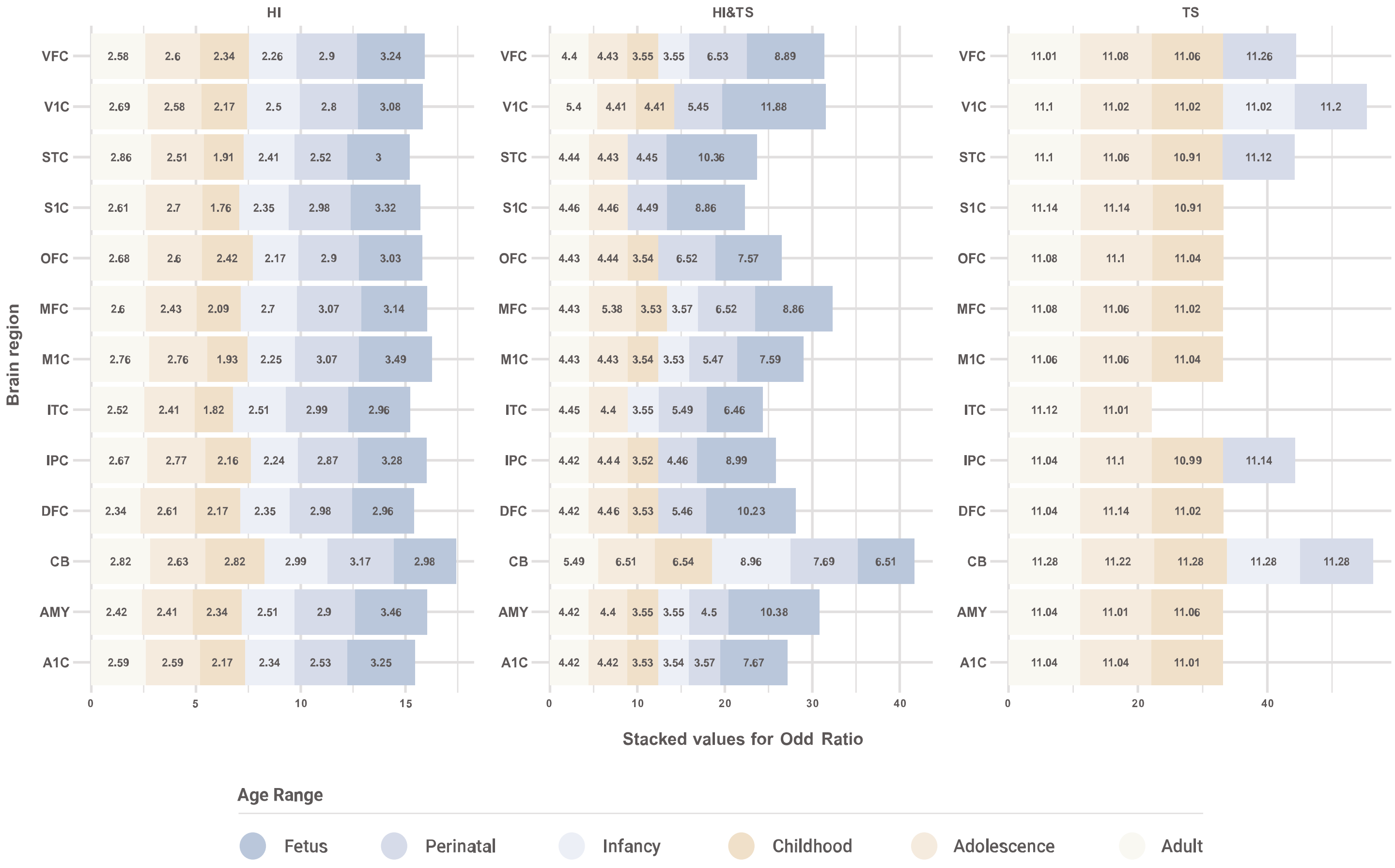

Supplemental Figure 4. Bar plot depicting Odds Ratios from significantly highly expressed qDSGs in key areas of the brain across all developmental stages.

Odd Ratios for qDSGs in each expression rank, i.e. high (A), medium (B) or low (C) expressed.

Supplemental Methods:

Meta-analysis methodology

The Gene SFARI Copy Number Variants (CNVs) database (https://gene.SFARI.org/database/cnv/) entails a collection of annotated genomic regions that includes information such as number of reported patients with NDDs and controls carrying deletions and/or duplications, ASD associated genes within these regions, genomic coordinates or detection platforms. Currently, there are two versions of this database: an archive version, more extensive, which includes CNVs reported throughout the entire genome and which do not necessarily have a significant relevance in autism, and an updated version, which only includes those regions that are highly significant in the autistic phenotype.

Data contained in SFARI website was extracted using a customized web scraper. Through this procedure, we obtained information of cases and controls, at the single patient level as well as the characteristics of the cohorts in which these probands were included.

Supplemental information table 1. Overview of data obtained from SFARI collection that were used in the statistical meta-analysis.

**Cases:** copy number variant carriers with an NDD diagnosis	Individual dataset<br>• Total initial number of carriers included in SFARI before the standardization: 23.907<br>• Total number of carriers after standardization procedure: 16.999<br>• Number of carriers included in our statistical analysis, after assessing cohort overlap and removing duplicates: 11,614
	Population dataset<br>• Initial number of studies included in SFARI: 565<br>• Studies included in the statistical test: 14<br>• Total population: 61,817
**Control:** copy number variant carriers without an NDD diagonisis.	Individual dataset<br>• Total initial number of carriers included in SFARI before the standardization: 6,949<br>• Total number of carriers after post-standardization procedure: 5,498<br>• Number of control carriers included in our statistical analysis, after assessing cohort overlap and removing duplicates: 4,031
	Population dataset<br>• Initial number of control studies included in SFARI: 105<br>• Studies included in the statistical test: 10<br>• Total population: 52,126

Standardization procedure

Bringing together formats and characteristics of different data fields under a common framework is a critical process in the study of large-scale cohorts. In particular, medical data can involve a high level of heterogeneity, due to the intrinsic complexity of biological processes and the disparity between techniques, years of study or even the healthcare facilities where analyses have been undertaken. Once

the SFARI data was obtained we detected several issues for downstream data processing (e.g., duplicate probands, inconsistencies in genomic coordinates reported or positions reported in different builds). To address data quality issues, we then proceeded to implement a data processing and standardization step, as detailed below divided by category of data intervention.

Patient Identifiers standardization

Individual patient identifiers standardization was crucial to carry out a reliable statistical analysis, since SFARI database includes duplicate entries both in cases and control samples. This is because numerous studies that were performed for the same region, based on the same case/control cohorts, are stored in SFARI database as separate entries. This fact is captured in the significant reduction that results, especially in cases, between the number of carriers before and after the standardization of the individual identifiers. (Supplemental table 1).

To resolve duplicates, we evaluated individually each of the papers of the studies from which the patients were obtained. Due to the high number of studies included, 565 case studies and 106 control studies, a first filtering was made selecting only those with more than 100 probands. This returned a total of 34 case studies and 13 control studies. We then reviewed all of them to identify the cohort used in their studies. To avoid overlapping cohorts, we selected those that did not share any patients among them either based on geographic location or because they came from different consortia.

Supplemental information table 2. Cohorts employed in this meta-analysis.

Study	PMID	Case Cohort Origin	Control Cohort Origin
**Yin CL , et al. (2016)**	27042285	Han Chinese	Han Chinese Cell and Genome Bank (HCCGB)
**Engchuan W , et al. (2015)**	25783485	AGP	SAGE (Study of Addiction Genetics and Environment), Ontario Colorectal Cancer study, HABC (Health Aging and Body Composition)
**Kaminsky EB , et al. (2011)**	21844811	ISCA	Portugal, UK and Sweden controls, PubMed: 19571809, PubMed: 19592680, PARC, NINDS, HGDP
**Nava C , et al. (2013)**	23632794	Centre de Référence déficiences intellectuelles de causes rares, Centre Diagnostic Autisme, Pitié-Salpêtrière Hospital, the Fondation Lejeune	NA
**Prasad A , et al. (2013)**	23275889	Canadian cohort	BioServe
**Nguyen LS , et al. (2013)**	23376982		
**Krumm N , et al. (2015)**	25961944	Simons Simplex Collection (SSC)	NA
**Kanduri C , et al. (2015)**	26052927	Finish cohort	Finish cohort
**Fitzgerald T , et al (2014)**	25533962	Decipher	Scottish Family Health Study
**Gazzellone MJ , et al. (2014)**	25170348	Children Development and Behavior Research Center (CDBRC)	Ontario Population Genomics Platform (OPGP), Han Chinese HapMap project, Han Chinese by Lou et al, Han Chinese Singapore Genome Variation Project
**Kushima I , et al. (2018)**	30208311	Japanese cohort	Japanese cohort
**Leppa VM , et al. (2016)**	27569545	AGRE	AGRE

**Quintela I , et al. (2017)**	28506748	Galician cohort	NA
**Girirajan S , et al. (2012)**	22970919	US cohort	NA
**Glessner JT , et al. (2009)**	19404257	NA	ACC
**Tropeano M , et al. (2013)**	23637818	NDD cohort	WTCCC2

Supplemental Information:

Shared etiology of NDDs [*1]

Several NDD-risk CNV loci have been reported to be associated with risk of more than one NDD. For instance, both deletion or duplication carriers at 16p11.2 have been found to exhibit ADHD, ID, ASD or epilepsy with significantly greater frequency than controls[1; 2]. Alternatively, it is estimated that individuals carrying monosomy in the 22q11.2 region are ~40% more likely to develop schizophrenic-spectrum disorders in adults[3], although it has also been observed that 10%-50% of patients with this same deletion[4], also known as Velocardiofacial/DiGeorge syndrome, report autism. Other behavioral disorders observed in affected individuals include attention deficit hyperactivity disorder, mood and anxiety disorders [4-6]

Other CNV loci associated with risk for both ASD and SCZ include deletions in 3q29 and 17q12, as well as duplications in 7q11.23 or 16p13.1 , in agreement with the previously reported shared etiology for these disorders[7-9].This finding also holds true for novel regions that we have detected, most of which also overlap with known risk regions. Among the newly identified regions we found some overlapping with previously known regions, such as 21q11.2-q22.3, 22q11.21-q11.23, which include the previously mentioned 21q11.2, or 8p23.3- p23.1, overlapping with 8p23.1. Few cases have been described for this terminal region of chromosome 8, and the most frequent clinical manifestations include ID[10; 11], ADHD[12] , ASD[13], and epilepsy[14]. Likewise, the overlapping regions 1p36.33-p36.22 and 1p36.33-p36.32[15; 16], 5q35.2-q35.3, or 6p terminal deletions (6p25.3, 6p25.3-p25.1)[17; 18] have also been associated with SZC, ID and ASD.

Details about other validated NDD-risk genes [*2]

In the 3q29 CNV loci we found PAK2, a candidate gene for which a high confidence level of association with NDD risk had not yet been established. However, it had been shown that this

serine/threorine kinase is essential in the regulation of cytoskeletal dynamics, and that knockout animal models recapitulated disturbed neurological synaptic patterns seen in patients with ASD[19]. In our analysis PAK2, a validated candidate gene, is closely associated with the MAPK stress activated cascade, an essential pathway in brain function, learning and memory. In iHART, a single variant has been found deleting this gene in four different families, and for two of which, all children diagnosed with ASD had this variant (Supplemental Table 3).

For CHM3, in the 1q43-44 region, cases with autism and ID have been previously reported[20]. In our analyses we not only validated its association to NDD-risk, but also found a set of clinical signs and symptoms in patients from Decipher with a statistically significant association to this NDD-risk gene: depressed nasal bridge, upslanted palpebral fissure, short neck, downturned corners of the mouth, sparse hair, micrognathia, epicanthus and hypoplasia of corpus callosum (Supplemental Table 3).

Supplemental references:

1. Niarchou, M., Chawner, S., Doherty, J.L., Maillard, A.M., Jacquemont, S., Chung, W.K., Green-Snyder, L., Bernier, R.A., Goin-Kochel, R.P., Hanson, E., et al. (2019). Psychiatric disorders in children with 16p11.2 deletion and duplication. Transl Psychiatry 9, 8.
2. Smith, H., Lane, C., Al-Jawahiri, R., and Freeth, M. (2022). Sensory processing in 16p11.2 deletion and 16p11.2 duplication. Autism Res 15, 2081-2098.
3. Schneider, M., Debbane, M., Bassett, A.S., Chow, E.W., Fung, W.L., van den Bree, M., Owen, M., Murphy, K.C., Niarchou, M., Kates, W.R., et al. (2014). Psychiatric disorders from childhood to adulthood in 22q11.2 deletion syndrome: results from the International Consortium on Brain and Behavior in 22q11.2 Deletion Syndrome. Am J Psychiatry 171, 627-639.
4. Ousley, O., Evans, A.N., Fernandez-Carriba, S., Smearman, E.L., Rockers, K., Morrier, M.J., Evans, D.W., Coleman, K., and Cubells, J. (2017). Examining the Overlap between Autism Spectrum Disorder and 22q11.2 Deletion Syndrome. Int J Mol Sci 18.
5. Bertran, M., Tagle, F.P., and Irarrazaval, M. (2018). Psychiatric manifestations of 22q11.2 deletion syndrome: a literature review. Neurologia (Engl Ed) 33, 121-128.
6. Hoeffding, L.K., Trabjerg, B.B., Olsen, L., Mazin, W., Sparso, T., Vangkilde, A., Mortensen, P.B., Pedersen, C.B., and Werge, T. (2017). Risk of Psychiatric Disorders Among Individuals With the 22q11.2 Deletion or Duplication: A Danish Nationwide, Register-Based Study. JAMA Psychiatry 74, 282-290.
7. Gandal, M.J., Haney, J.R., Parikshak, N.N., Leppa, V., Ramaswami, G., Hartl, C., Schork, A.J., Appadurai, V., Buil, A., Werge, T.M., et al. (2019). Shared Molecular Neuropathology Across Major Psychiatric Disorders Parallels Polygenic Overlap. Focus (Am Psychiatr Publ) 17, 66-72.
8. Kushima, I., Aleksic, B., Nakatochi, M., Shimamura, T., Okada, T., Uno, Y., Morikawa, M., Ishizuka, K., Shiino, T., Kimura, H., et al. (2018). Comparative Analyses of Copy-Number Variation in Autism Spectrum Disorder and Schizophrenia Reveal Etiological Overlap and Biological Insights. Cell Rep 24, 2838-2856.
9. Wu, X., Huai, C., Shen, L., Li, M., Yang, C., Zhang, J., Chen, L., Zhu, W., Fan, L., Zhou, W., et al. (2021). Genome-wide study of copy number variation implicates multiple novel loci for schizophrenia risk in Han Chinese family trios. iScience 24, 102894.
10. Hutchinson, R., Wilson, M., and Voullaire, L. (1992). Distal 8p deletion (8p23.1----8pter): a common deletion? J Med Genet 29, 407-411.
11. Wu, Y., Ji, T., Wang, J., Xiao, J., Wang, H., Li, J., Gao, Z., Yang, Y., Cai, B., Wang, L., et al. (2010). Submicroscopic subtelomeric aberrations in Chinese patients with unexplained developmental delay/mental retardation. BMC Med Genet 11, 72.
12. Shi, S., Lin, S., Chen, B., and Zhou, Y. (2017). Isolated chromosome 8p23.2-pter deletion: Novel evidence for developmental delay, intellectual disability, microcephaly and neurobehavioral disorders. Mol Med Rep 16, 6837-6845.
13. Chien, W.H., Gau, S.S., Wu, Y.Y., Huang, Y.S., Fang, J.S., Chen, Y.J., Soong, W.T., Chiu, Y.N., and Chen, C.H. (2010). Identification and molecular characterization of two novel chromosomal deletions associated with autism. Clin Genet 78, 449-456.
14. Nucaro, A., Pisano, T., Chillotti, I., Montaldo, C., and Pruna, D. (2011). Chromosome 8p23.2-pter: a critical region for mental retardation, autism and epilepsy? Clin Genet 79, 394-395; author reply 396.
15. Guo, S., Liu, J., Li, W., Yang, Y., Lv, L., Xiao, X., Li, M., Guan, F., and Luo, X.J. (2021). Genome wide association study identifies four loci for early onset schizophrenia. Transl Psychiatry 11, 248.

16. Radio, F.C., Pang, K., Ciolfi, A., Levy, M.A., Hernandez-Garcia, A., Pedace, L., Pantaleoni, F., Liu, Z., de Boer, E., Jackson, A., et al. (2021). SPEN haploinsufficiency causes a neurodevelopmental disorder overlapping proximal 1p36 deletion syndrome with an episignature of X chromosomes in females. Am J Hum Genet 108, 502-516.
17. Bogani, D., Willoughby, C., Davies, J., Kaur, K., Mirza, G., Paudyal, A., Haines, H., McKeone, R., Cadman, M., Pieles, G., et al. (2005). Dissecting the genetic complexity of human 6p deletion syndromes by using a region-specific, phenotype-driven mouse screen. Proc Natl Acad Sci U S A 102, 12477-12482.
18. Rraku, E., Kerstjens-Frederikse, W.S., Swertz, M.A., Dijkhuizen, T., van Ravenswaaij-Arts, C.M.A., and Engwerda, A. (2023). The phenotypic spectrum of terminal and subterminal 6p deletions based on a social media-derived cohort and literature review. Orphanet J Rare Dis 18, 68.
19. Wang, Y., Zeng, C., Li, J., Zhou, Z., Ju, X., Xia, S., Li, Y., Liu, A., Teng, H., Zhang, K., et al. (2018). PAK2 Haploinsufficiency Results in Synaptic Cytoskeleton Impairment and Autism-Related Behavior. Cell Rep 24, 2029-2041.
20. Khadija, B., Rjiba, K., Dimassi, S., Dahleb, W., Kammoun, M., Hannechi, H., Miladi, N., Gouider-Khouja, N., Saad, A., and Mougou-Zerelli, S. (2022). Clinical and molecular characterization of 1q43q44 deletion and corpus callosum malformations: 2 new cases and literature review. Mol Cytogenet 15, 42.
