# Supplementary material for: Identification of novel driver risk genes in CNV loci associated with neurodevelopmental disorders
Source: HGG Adv. 2024 Jun 6;5(3):100316. doi: 10.1016/j.xhgg.2024.100316 (PMC11264174; doi:10.1016/j.xhgg.2024.100316)
Supplement: Document S2. Article plus supplemental information [file mmc3.pdf]

HGG Advances

ARTICLE

Identification of novel driver risk genes in CNV loci associated with neurodevelopmental disorders

Sara Azidane,[1,2] Xavier Gallego,[1] Lynn Durham,[1] Mario Cáceres,[2,3,4] Emre Guney,[1] and Laura Pérez-Cano[1,5,*]

Summary

Copy-number variants (CNVs) are genome-wide structural variations involving the duplication or deletion of large nucleotide sequences. While these types of variations can be commonly found in humans, large and rare CNVs are known to contribute to the development of various neurodevelopmental disorders (NDDs), including autism spectrum disorder (ASD). Nevertheless, given that these NDD-risk CNVs cover broad regions of the genome, it is particularly challenging to pinpoint the critical gene(s) responsible for the manifestation of the phenotype. In this study, we performed a meta-analysis of CNV data from 11,614 affected individuals with NDDs and 4,031 control individuals from SFARI database to identify 41 NDD-risk CNV loci, including 24 novel regions. We also found evidence for dosage-sensitive genes within these regions being significantly enriched for known NDD-risk genes and pathways. In addition, a significant proportion of these genes was found to (1) converge in protein-protein interaction networks, (2) be among most expressed genes in the brain across all developmental stages, and (3) be hit by deletions that are significantly over-transmitted to individuals with ASD within multiplex ASD families from the iHART cohort. Finally, we conducted a burden analysis using 4,281 NDD cases from Decipher and iHART cohorts, and 2,504 neurotypical control individuals from 1000 Genomes and iHART, which resulted in the validation of the association of 162 dosage-sensitive genes driving risk for NDDs, including 22 novel NDD-risk genes. Importantly, most NDD-risk CNV loci entail multiple NDD-risk genes in agreement with a polygenic model associated with the majority of NDD cases.

Introduction

Copy-number variants (CNVs) are genome-wide structural changes involving the duplication or deletion of large nucleotide sequences, which can range from one kilobase (kb) to several megabases (Mb). This type of variation is commonly found in humans, and, even though it has been shown that most large CNVs are subject to high purifying selection,[1] previous reports have suggested that between 5% and 10% of the human genome is affected by CNVs.[2]

These variants could have significant functional consequences and they have been identified as an important mechanism of evolutionary adaptation. Rare genetic material gains or losses are also known to contribute substantially to the development of various neurodevelopmental disorders (NDDs), including autism spectrum disorder (ASD). Rare chromosomal rearrangements have been found in up to 10% of individuals with ASD,[3,4] and it has additionally been observed that a larger fraction of the genome of these individuals is affected by CNVs compared with neurotypical individuals.[5,6]

In particular, both rare *de novo* and inherited CNVs have been identified as penetrant variants that confer a major risk in the development of NDDs.[3] Identification of such CNVs requires exceptionally large cohorts[1,7] in genome-wide analysis, which are not often available, to reach sufficient statistical power to detect the association of loci with NDD susceptibility. Despite such low frequency of rare CNVs, certain NDD-risk loci have been recurrently identified,[8–10] most notably 1q21.1, 3q29, 7q11.23, 15q11-q13, 15q13.3, 16p11.2, 5q35, 17q12, and 22q11.2. These risk loci can usually be affected by copy-number gains or losses (Table S2), most of which have been independently associated with the manifestation of various NDDs. For instance, both 16p11.2 deletion and duplications confer 10-fold increase for ASD-risk,[10] while 16p11.2 duplications also show >10-fold increase of risk of schizophrenia.[9]

There are significant limitations that condition the study of the impact of these structural variants in NDDs. First, considering the nature of rare CNVs, it is expected that they affect broad regions of the genome, often spanning several genes. Indeed, this fact makes it particularly challenging to perform fine mapping and to pinpoint the critical genes responsible for the manifestation of the phenotype. Furthermore, the current paradigm of CNV pathogenicity is primarily oriented toward finding a single gene responsible for causing the disease. These pathogenicity models can explain diseases with minimal phenotypic variability (i.e., similar or nearly identical clinical features and symptoms among affected individuals) or with highly penetrant variants, as in the case of *RAI1* in Smith-Magenis syndrome, for example. But in the case of

[1]STALICLA Discovery and Data Science Unit, World Trade Center, Moll de Barcelona, Edif Este, 08039 Barcelona, Spain; [2]Institut de Biotecnologia i de Biomedicina, Universitat Autònoma de Barcelona, Bellaterra, 08193 Barcelona, Spain; [3]ICREA, 08010 Barcelona, Spain; [4]Research Programme on Biomedical Informatics (GRIB), Hospital del Mar Research Institute, Barcelona, Spain
[5]Lead contact
*Correspondence: laura.perez-cano@stalicla.com
https://doi.org/10.1016/j.xhgg.2024.100316.
© 2024 This is an open access article under the CC BY-NC-ND license (http://creativecommons.org/licenses/by-nc-nd/4.0/).

complex diseases such as most neurodevelopmental conditions, these monogenic models have proven unsuccessful.

Due to the inherent degree of complexity in the study of such risk variants, not only are the causative genes often difficult to identify, but also their role in the pathophysiology underlying the manifestation of the disorder is difficult to characterize. Prior literature shows that pathogenic CNVs can give rise to a certain clinical phenotype through different events, either by affecting regulatory regions of the genome, by disrupting genes, or by affecting gene dosage.[11] However, there is growing evidence that the main mechanism whereby pathogenic CNVs trigger the phenotype is by means of dose sensitivity. To function properly, genes in risk regions that are sensitive to these dosage perturbations (i.e., haploinsufficient [HI] and/or triplosensitive [TS] genes) might require to be in stoichiometric equilibrium with other genes to ensure proper protein assembly, to form aggregates at high concentration levels, or to comply with concentration thresholds.[12] Therefore, when a copy-number gain or loss overlaps with any of these HI and/or TS genes and changes their dosage, the function of such gene is disrupted, resulting in the manifestation of the disease. Besides, previous studies have shown that the reciprocal genomic disorders, i.e., when the same region is associated to an NDD by both deletion or duplication, often show "mirror phenotypes." In this way, duplication of the region causes the opposite phenotype of what would be observed by the deletion, suggesting that one or more genes in each region might indeed be causing the phenotype through a dosage sensitivity mechanism.

In this study, we performed a meta-analysis of CNVs called from 11,614 affected individuals with NDDs and 4,031 neurotypical control individuals from the SFARI gene database to identify NDD-risk regions and the causative dosage-sensitive genes within these broad regions.

Subjects and methods

Meta-analysis for the identification of NDD-risk loci

We carried out a statistical meta-analysis of CNV calls covering 2,273 genomic loci in 11,614 affected individuals with NDDs and 4,031 control individuals from 18 studies (14 case studies and 4 additional control studies) gathered under the SFARI gene website. The data were standardized to avoid duplicated samples and to include only data derived from array analysis (see supplemental methods).

The statistical procedure of this case-control study entailed a two-sided Fisher's exact test in which a 2 × 2 contig table is created for each of the 2,273 genomic regions analyzed, based on the number of cases and control individuals with and without CNVs in the given area of the genome. In this way, the degree of association between the presence of a CNV in that region and the expression of the phenotype is evaluated. To correct for multiple hypothesis testing, the p values were adjusted using Bonferroni correction and associations called statistically significant if $p < 2.19 \times 10^{-5}$ (i.e., below adjusted $p = 0.05$).

Dosage sensitivity

To identify which dosage-sensitive genes in the NDD-risk loci lead the manifestation of the phenotypes, we evaluated all genes whose coding sequence fully overlapped with the pathogenic risk region and annotated them with scores for probability of haploinsufficiency (pHI) and the probability of triplosensitivity (pTS). These scores were recently described by Collins et al.[8] and provide a more precise metric of genes intolerant to dosage alterations than those used for short variants. The validity of these scores, which range from 0 to 1, has been investigated by assessing their accuracy in already known dosage-sensitive genes ($p < 10^{-100}$, two-sided Kolmogorov-Smirnov test of pTS and pHI scores).[8] Cutoffs to define haploinsufficiency and triplosensitivity in these genes were established by Collins et al.[8] at pHI $\geq$ 0.84 and pTS $\geq$ 0.993, taking into consideration that the effects of deleted regions are typically stronger than those of duplications.

We therefore generated a catalog of qualifying dosage-sensitive risk genes (qDSGs), by including dosage-sensitive genes located within our previously defined NDD-risk CNV loci. We categorized these qDSGs into three different tiers: HI genes (with pHI $\geq$ 0.840 and pTS $\leq$ 0.993), TS genes (with pTS $\geq$ 0.993 and pHI $\leq$ 0.840), and genes that are both haploinsufficient and triplosensitive (bHITS) (with pTS $\geq$ 0.993 and pHI $\geq$ 0.84).

To assess whether our list of qDSGs was enriched with genes previously identified as NDD-risk genes, we employed the GeneTrek catalog of NDD-risk genes. In this gene catalog high-confidence genes are extracted from SPARK/SFARI category 1, SysID primary, G2P DD "confirmed," and "Brain|Cognition," as well as DBD tier1 and AR categories. On the other hand, candidate genes are derived from SPARK/SFARI categories 2, 3, and S, the SysID candidates, the DBD tier 2 to 4, the G2P DD others than categories "confirmed" or "Brain|Cognition."[13]

Gene ontology enrichment analysis

To assess the biological implications at the level of function and disease mechanism, we performed a gene set enrichment analysis of the dosage-sensitive genes found in the NDD-risk CNV regions. For this, we used g:Gost from g:Profiler (version e109_eg56_p17_1d3191d), a suitable tool for checking enrichment of unranked gene lists.[14] Gene ontology (GO) (release 2023-03-06) was used as the reference gene set database, which provides thousands of standardized terms classified in biological processes, cellular components, and molecular functions.[15,16] As a result of this analysis, we obtained lists of enriched (i.e., overrepresented) terms associated with our three sets of dosage-sensitive genes, namely TS genes, HI genes, or bHITS genes. Bonferroni correction was used to adjust multiple hypothesis testing and terms below an adjusted p value of 0.05 were deemed significantly associated to the genes provided as input.

TDT analysis

To evaluate how rare deletions affecting genes in our catalog are transmitted, we analyzed multiplex family CNV data from the iHART cohort, extracted from Ruzzo et al.,[17] to perform a transmission disequilibrium test (TDT). The CNV callers utilized to detect variants in the iHART cohort (SMuFin, GenomeSTRiP, BreakDancer, LUMPY) exhibited lower sensitivity in identifying duplications and only rare deletions were used in this analysis.[17] We performed a one-sided binomial test that evaluates if the transmission rate of inherited deletions in HI genes within qDSGs deviate significantly from the expected Mendelian transmission

rate (i.e., ~50%), considering all the siblings with ASD within each family. This approach yields a statistical p value that determines whether there is a significant over transmission of CNVs in our risk genes from parents to children with an ASD diagnosis.

Identification of dosage-sensitive genes associated with NDD-risk

A two-sided Fisher's test was used to infer the association of each qDSGs with risk for NDDs. In particular, we compared the frequency of affected individuals with NDDs from iHART[17] and Decipher[18] carrying rare deletions and duplications hitting the gene, against the frequency of neurotypical control individuals from the 1000 Genomes from the DGV[19] database carrying such variation within the gene. Rare variants were defined as those not overlapping more than 50% with common variants (AF $\geq$ 0.001) in the gnomAD database.[20] It is important to note that, when evaluating deletions, we compared the frequency of rare deletions in both iHART and Decipher affected individuals with that in the 1000 Genomes control individuals. However, for duplications, our analysis exclusively utilized data from Decipher affected individuals, due to the lack of sensitivity of the calling methods used to identify duplications in the iHART cohort. A Bonferroni-based multiple test correction was applied, with significance stablished at 1.93×10^{-4} for HI genes, 8.33×10^{-3} for TS genes, and 1.66×10^{-3}.

Brain-specific gene expression analysis

To better grasp how our qDSGs behave in the developing brain over time, we analyzed data from the Brain Span atlas database, which contains gene expression data from typical brain development. We made the RNA-seq counts more comparable by utilizing a variance stabilizing transformation,[21] which helps to correct for differences in gene expression levels. We then identified genes that were among the top 5% in terms of expression during various developmental stages (fetal, perinatal, infancy, childhood, adolescence, and adulthood) and in different brain regions (such as neocortex, amygdala, and cerebellum). We then conducted several two-sided Fisher's exact tests to assess whether there was enrichment of our qDSGs in the top 5% expressed genes at different developmental stages and in different areas of the brain.

Protein-protein interaction network

The broad spectrum of NDDs involves a lattice of complex interactions of the proteins encoded by the risk genes. To understand the functional modules that can be established between the genes in the previously defined NDD-risk regions, we built a protein-protein interaction (PPI) network using the STRING platform, providing a protein-protein connection in the network for interactions with an interaction score value $\geq$0.400, considering only high-confidence PPIs as defined by experiments and/or databases.

To investigate the biological significance of the connectedness of nodes in the PPI network, the expected and observed numbers of edges between specific groups of genes were evaluated. The expected number of edges is the number of connections that would be observed if the selection of nodes were random, while the observed is the actual number of connections within the network. If the observed number of edges is significantly higher than the expected number of connections, it indicates a higher interconnectivity between the proteins in the network, pointing to a non-random organization of the genes of interest.

Association of clinical phenotypes with dosage-sensitive genes

We conducted a statistical analysis to infer significant gene-phenotype relationships by analyzing clinical data from 3,708 affected individuals with NDDs in the Decipher[18] public database. We calculated the incidence frequencies of each phenotype per gene (number of affected individuals with a certain clinical phenotype and a CNV overlapping a specific gene divided by the total of affected individuals with a CNV affecting such a gene) and compared them with the phenotype frequencies for the Decipher population of affected individuals with NDDs. We then selected those phenotypes whose frequency of incidence in the gene was higher than the frequency in the general population and performed a two-sided Fisher's test to infer whether each gene-phenotype association was statistically significant. By using Bonferroni correction, significance association threshold was established a p value below 1.22×10^{-6}.

Results

Identification of 23 novel NDD-risk CNV loci

Out of the 2,273 genomic loci (also known as regions) with CNV calls analyzed from the SFARI gene database in our meta-analysis, 180 were statistically significantly overrepresented in individuals with NDDs ($p < 0.05$) with an odds ratio higher than 1, and 41 of those passed Bonferroni threshold to strictly correct for multiple testing (Table S1). We hence determined these 41 regions as high-confidence NDD-risk loci. To validate and assess which of these CNV loci were novel, significantly associated regions from our analysis were compared with the ones previously reported by widely recognized reference studies (Sanders et al. 2015[10], Collins et al.,[8] and regions recognized as relevant in SFARI database). These studies have identified a set of 50 NDD-risk (Table S2) regions after analyzing CNV data from thousands of affected individuals with NDDs and control individuals.

Out of these 41 regions, 24 were novel. These included 19 not previously reported genomic loci as well as 5 loci expanding and/or overlapping with previously known NDD-risk regions (Table S1). For instance, the 8p23.1-p23.3 genomic locus reported in our analysis as a novel region associated to NDDs comprises a large region that expands the 8p23.1 region included in the SFARI NDD-risk CNV catalog, as well as 8p23.2 and 8p23.3 regions associated with NDDs in the Collins et al.[8] study.

Nevertheless, Bonferroni multiple test correction approach is strict in the identification of region-disorder associations. Considering the signal from regions that do not reach the Bonferroni threshold but pass nominal significance (with $p < 0.05$ and odds ratio >1), we identified 11 additional previously reported risk regions (please see Table S2). Importantly, if we examine a subset of 12 high-confidence NDD-risk regions that were previously reported in at least two studies (i.e., 3q29, 7q11.23, 15q11.2-q13.1, 15q13.2-q13.3, 16p11.2, 22q11.21, 1q21.1, 2p16.3, 15q13.3, 16p12.2, 16p13.3, and 17q12)

Table 1. Number of dosage-sensitive genes located within all/novel NDD-risk regions that are also included in the SFARI catalog of ASD-risk genes

SFARI gene score	No. of qDSGs in SFARI catalog out of total qDSGs	No. of qDSGs in SFARI catalog out of total qDSGs in novel regions
1	25	18
2	14	10
3	14	9
S	10	8
Total	63/294 total qDSGs (57 HI/0 TS/6 TS + HI (258 HI/6 TS/30 TS + HI)	45/197 total qDSGs (40 HI/0 TS/5 TS + HI) (172 HI/4 TS/21 TS + HI)

Score 1 defines high confidence genes, while score 2 labels defines genes with strong association to the phenotype. Score 3 genes present little evidence of association and S genes are considered syndromic.

we identified all of them in our meta-analysis, 11 surpassing the Bonferroni correction threshold, and 1 with nominal significance ($p < 0.05$).

NDD-risk CNV loci are significantly enriched for dosage-sensitive risk genes

We then evaluated the haploinsufficiency and triplosensitivity of genes located in the 41 high-confidence NDD-risk regions identified. Of the 2,097 genes enclosed in these regions, we found a total of 294 qDSGs, from which 258 were HI (pHI > 0.84), 6 were TS (pTS > 0.993), and 30 were both TS and HI (pTS > 0.993 and pHI > 0.840). When limiting the scope of the evaluation to the 1,256 genes located in the 24 novel regions, we could identify 172 HI genes (pHI > 0.840), 4 showing triplosensitivity (pTS > 0.993) and 21 that were both TS and HI (pTS > 0.993 and pHI > 0.840), totaling 197 qDSGs.

Interestingly, a statistically significant overrepresentation of both HI ($p = 7.32 \times 10^{-3}$) and TS ($p = 1.33 \times 10^{-6}$) genes was found in the 41 NDD-risk regions, although no enrichment was observed for genes showing both haploinsufficiency and triplosensitivity. Similarly, we detect enrichment of HI genes ($p = 4.25 \times 10^{-2}$) and TS genes ($p = 7.31 \times 10^{-5}$) in the novel NDD-risk regions, but no enrichment for bHITS genes was observed either ($p = 2.48 \times 10^{-1}$).

Among the 258 genes that were identified as HI within the NDD-risk regions, 57 HI genes also appear in the SFARI catalog of ASD-risk genes. From the group of 172 HI genes in novel regions, 40 HI genes are also reported as ASD-risk in SFARI (Table 1; Figure 1). This overlap implies a statistically significant overrepresentation of ASD-risk SFARI genes among HI genes in both novel ($p = 2.74 \times 10^{-4}$) and all NDD-risk regions ($p = 4.6 \times 10^{-5}$) identified in this study. We also performed an enrichment analysis with genes reported in recent large-scale ASD-risk SNP association studies to evaluate, from an orthogonal perspective, the enrichment of known ASD-risk genes in our list of qDSGs. We employed the genes reported in two different studies, Satterstrom et al.[22] and Trost et al.,[23] and also detected an enrichment of ASD-risk genes in the list of qDSGs ($p = 1.72 \times 10^{-4}$) and also in the subset of those genes that are in novel CNV regions reported in this study ($p = 3.68 \times 10^{-3}$).

On the other hand, we sought to ascertain whether a broader list of known NDD-risk genes was significantly enriched for our qDSGs. Using a recent extensive catalog of genes associated with NDDs (GeneTrek) we could observe that out of the 294 qDSGs, 259 appear in this catalog as candidate NDD-risk genes ($p = 1.25 \times 10^{-64}$), and from the subset of 197 qDSGs found in novel regions, 173 also overlap with this catalog ($p = 5.59 \times 10^{-43}$; Table 2).

Validation of 22 novel NDD-risk qDSGs

To provide further validation of our qDSG list in terms of their association with NDD-risk, we examined whether these genes were also significantly affected by CNVs in affected individuals with NDDs from independent cohorts. To this end, we first conducted a TDT and observed that deletions fully overlapping with HI genes within qDSGs were transmitted to offspring with autism more than expected by chance in 493 multiplex ASD families from the iHART dataset, including both maternally ($p = 4.07 \times 10^{-5}$, probability of success = 0.726 [95% CI, 0.618–0.818]) and paternally ($p = 6.3 \times 10^{-3}$, probability of success = 0.666 [95% CI, 0.545–0.773]) inherited deletions.

We then conducted a burden analysis by performing a Fisher's test using 4,281 affected individuals with NDDs from iHART and Decipher and 2,504 control individuals from the 1000 Genomes cohort, where only rare deletions and duplications from these subjects (with an allelic frequency < 0.1%) were considered. This analysis provided evidence for the association of 162 genes out of 294 qDSGs with risk for NDDs (Bonferroni correction and odds ratio > 1), of which 140 qDSGs were previously described as high confidence or candidate NDD-risk genes and 22 are novel risk genes (Figure 2).

On the other hand, a total of 56 out of the 132 qDSGs for which we did not find association with NDD-risk (i.e., not surpassing multiple test correction threshold) exhibited an association trend with a nominal p value below 0.05 (Table S3).

Implication of qDSGs in biological processes related to neuronal development and function

To assess whether qDSGs may be involved in biological processes essential for brain development and/or function,

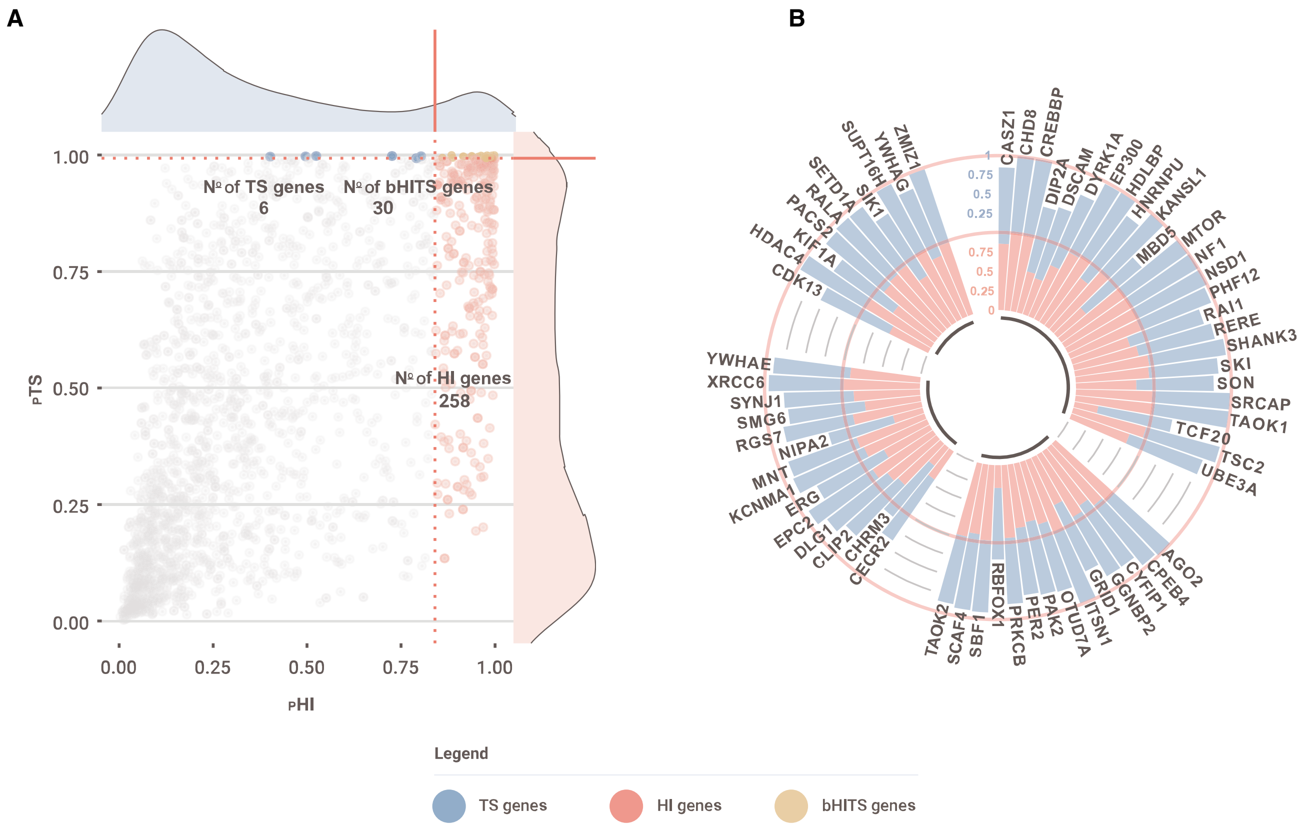

Figure 1. Dosage-sensitive genes in NDD-risk regions overlap with ASD-risk genes
Scatterplot A shows the dose sensitivity score values (pHI and pTS) for genes in regions identified as NDD-risk. Gray dots in the scatterplot stand for genes that do not meet the threshold to be considered HI genes, TS genes, or bHITS genes. Depicted in red are the genes that meet the threshold for haploinsufficiency, HI genes, and in blue the ones that meet the threshold for triplosensitivity, TS genes. Genes that surpass the threshold for both haploinsufficiency and triplosensitivity, bHITS genes, are depicted in yellow in the upper right quadrant of the grid. The circular bar plot (B) represents the 63 dosage-sensitive genes that are present in both our list of qDSGs and the SFARI curated catalog of ASD-risk genes, according to the degree of evidence of association.

we obtained functional information from the enrichment of GO terms, i.e., over-represented terms for each of the three gene lists according to its dosage-sensitive type.

We identified a total of 384 unique GO terms significantly (p value adjusted after Bonferroni correction < 0.05) enriched in qDSGs (Figure 3; Table S4). Taking into account genes in novel NDD-risk regions, we found 297 significantly enriched GO terms in qDSGs enclosed in those regions (Table S5). In both cases strong statistical enrichment is observed for terms such as neuronal morphology, synapses, signaling pathways, and control of gene expression (Figures 3A–3C). This association is observed at the level of cellular components, molecular functions, and biological processes. For example, of the 391 terms related to qDSGs in all NDD-risk regions, 39 GO terms refer to synaptic processes associated with 64 qDSGs, of which 2 are novel NDD-risk candidate genes, while 19 are known high-confidence genes, i.e., with an SFARI score 1 or present in the high confidence curated list of NDD-risk genes mentioned above. We also found 29 GO terms associated with morphology or development of neuronal elements such as dendrites and axons involving 57 qDSGs, of which 54 had been previously associated with NDDs by reference catalogs. The identification of enriched terms associated with chromatin regulatory processes is in agreement with previous literature reporting the crucial role of chromatin remodelers during corticogenesis, cell migration, and cell-type specification.[24,25] In particular, we have found 44 GO terms associated with processes related to histone and chromatin modification, as well as DNA binding, associated with 80 genes from our dosage-sensitive gene list, of which 9 are novel NDD-risk candidate genes not described before.

Importantly, we performed the same GO enrichment analysis using all 1,031 genes from the curated SFARI catalog and found a significant overlap between terms enriched for SFARI genes and those identified from our qDSG list, including a significant overlap for terms related to HI genes ($p = 8.14 \times 10^{-292}$; Figure 3D), for TS genes ($p = 1.26 \times 10^{1-8}$; Figure 3E) and for bHITS genes ($p = 6.56 \times 10^{-46}$; Figure 3F).

qDSGs are among the highest-expressed genes in brain along all developmental stages

Previous studies in affected individuals with NDD have detected aberrant expression of genes involved in pathways enriched in the GO analysis (synaptic formation, dendrite

Table 2. NDD-risk regions identified through our meta-analysis and the 294 corresponding qDSGs encompassed in them according to their dosage sensitivity type

CNV	Both TS and HI	HI	TS	CNV type	Chromosome
1p36.33-p36.22 (novel)	*CAMTA1***+, *MTOR***+	*SPSB1*+, *TARDBP**+, *SKI***+, *LRRC47*+, *TP73***+, *ICMT*+, *PARK7**+, *TMEM201**+, *CLSTN1**+, *PIK3CD**+, *UBE4B**+, *KIF1B**+, *PGD**+, *MFN2**+, *GNB1***+, *AJAP1**+, *CHD5***+, *DNAJC11**+, *RERE***+, *CASZ1***+, *PRDM16**+	N/A	deletion	1
1p36.33-p36.32 (novel)	N/A	*SKI***+, *LRRC47*+, *TP73***+, *GNB1***+, *AJAP1**+, *PRDM16**+	N/A	deletion	1
1q21.1	N/A	*TXNIP*, *PIAS3**	N/A	deletion-duplication	1
1q43-q44	*AKT3***+	*HEATR1**, *DESI2*+, *HNRNPU***+, *AHCTF1**+, *ZNF496**+, *ACTN2**, *RYR2*, *CHRM3**, *ZBTB18***+, *KIF26B**, *FMN2***, *RGS7**	N/A	duplication	1
2q23.1 (novel)	N/A	*MBD5***, *EPC2**	N/A	deletion-duplication	2
2q37.3 (novel)	*HDLBP***+	*COPS8*+, *COL6A3**+, *ILKAP**+, *PER2**+, *SNED1**+, *KIF1A***+, *HDAC4***+	N/A	deletion-duplication	2
3q22.1 (novel)	PLXND1*	*TMCC1*, *PIK3R4*, *CDV3*, *TOPBP1**, *RAB6B**, *ATP2C1**, *DNAJC13**, *TMEM108**	N/A	deletion-duplication	3
3q29	N/A	*HES1*+, *ATP13A3**, *UBXN7**+, *PAK2**+, *RPL35A**, *OPA1**, *ACAP2**+, *SENP5**+, *DLG1**+	N/A	deletion-duplication	3
4p16.3 (novel)	*CTBP1***+	*PCGF3**+, *FAM193A**+, *ADD1**+, *HTT***+	N/A	deletion-duplication	4
5q35.2-q35.3 (novel due to novel region boundaries)	*HNRNPH1***+, *CANX**+, *UNC5A**+	*NSD1***, *DBN1**, *FAF2**, *HNRNPAB**, *MAML1**, *MGAT1**, *TRIM41**, *CPEB4**, *FLT4**, *ADAMTS2**	N/A	duplication	5
6p25.3 (novel)	N/A	*FOXF2*+, *IRF4**+, *GMDS**	N/A	deletion-duplication	6
6p25.3-p25.1 (novel)	N/A	*NRN1**+, *FOXF2*+, *CDYL**, *IRF4**+, *PRPF4B**+, *SLC22A23*, *GMDS**	N/A	deletion	6
7p14.1 (novel)	N/A	*GLI3***, *RALA***, *INHBA**, *PSMA2**, *CDK13***	N/A	deletion-duplication	7
7q11.23	N/A	*BAZ1B**+, *LIMK1**+, *HIP1**, *YWHAG***, *CLIP2**+, *RSBN1L*, *PTPN12**	N/A	deletion-duplication	7
8p23.1	N/A	*TNKS**+, *XKR6**+, *LONRF1*+, *ANGPT2*+	N/A	deletion-duplication	8
8p23.3-p23.1 (novel due to novel region boundaries)	N/A	*TNKS**+, *XKR6**+, *LONRF1*+, *ANGPT2*+	N/A	deletion	8
8q24.3 (novel)	*AGO2***+, *PUF60***+	*PTK2**, *FAM83H*, *RPL8**, *SCRIB**	*CYHR1*+	deletion-duplication	8
10q22.3-q23.2 (novel)	N/A	*KCNMA1***, *RPS24**, *TSPAN14**, *CCSER2*, *DLG5**, *ZCCHC24**, *ZMIZ1***, *BMPR1A*, *GRID1**	N/A	deletion-duplication	10
14q11.2 (novel)	*ZNF219**+, *SUPT16H***+, *CHD8***+, *ACIN1**	*PRMT5**, *MMP14**, *TOX4**	*JPH4**	deletion-duplication	14
14q32.33	*MTA1**+	*INF2**+, *AKT1**+, *CEP170B**+, *JAG2**+, *KLC1**+, *PACS2***+, *PPP1R13B**+	N/A	deletion-duplication	14
15q11.2 (novel due to novel region boundaries)	N/A	*UBE3A***+, *CYFIP1**+, *NIPA2**+	N/A	deletion-duplication	15
15q11.2-q13.1	N/A	*UBE3A***+, *CYFIP1**+, *NIPA2**+, *TJP1**+	N/A	duplication	15
15q13.2-q13.3	N/A	*OTUD7A**+	N/A	deletion-duplication	15

(*Continued on next page*)

Table 2. *Continued*

CNV	Both TS and HI	HI	TS	CNV type	Chromosome
15q13.3	N/A	*OTUD7A*+*	N/A	deletion-duplication	15
16p11.2	*FBXL19**	*FBRS*, FUS*, SH2B1*+, TAOK2*+, TBC1D10B*, SRCAP**, SETD1A**, ATXN2L*+*	*MAZ*+, STX1B***	deletion-duplication	16
16p12.1 (novel)	N/A	*RBBP6*, ARHGAP17, IL21R*, TNRC6A*, GTF3C1*, XPO6**	N/A	deletion-duplication	16
16p12.2-p11.2 (novel due to novel region boundaries)	*FBXL19**	*PLK1*, FBRS*, FUS*, RBBP6*, ARHGAP17, IL21R*, SH2B1*+, TAOK2*+, TBC1D10B*, SRCAP**, SETD1A**, TNRC6A*, GTF3C1*, XPO6*, ATXN2L*+, PRKCB*, USP31*	*MAZ*+, STX1B***	duplication	16
16p13.11	N/A	*MYH11+*	N/A	deletion-duplication	16
16p13.3	*MAPK8IP3**+*	*RPS2*, CAPN15**+, TSC2**, CASKIN1*, TFAP4*, GLYR1*, UBN1*, ADCY9*, CREBBP**+, RBFOX1*+*	*UBE2I*+*	deletion-duplication	16
17p11.2	N/A	*MPRIP*+, COPS3*+, RAI1**+, ALKBH5*+, EPN2+, USP22*+*	*SREBF1+*	deletion-duplication	17
17p13.3 (novel)	*PRPF8*+, CLUH*+*	*NXN*+, ABR*+, YWHAE*+, PITPNA*+, MNT*+, PAFAH1B1**+, CRK*+, RTN4RL1*+, METTL16+, RAP1GAP2*+, SMG6*+*	N/A	deletion-duplication	17
17q11.2	*NLK*, GIT1*, SUPT6H*, NF1**+*	*TRAF4*, SSH2*, PHF12**, NUFIP2*, ATAD5*, RHOT1, PSMD11*, MYO18A*, TAOK1***	N/A	deletion-duplication	17
17q12	*ACACA*+, PIP4K2B**	*AP2B1*, GGNBP2*+, MLLT6*, PCGF2**, SYNRG*+, SRCIN1*, MED1*, CDK12*, SOCS7*, FBXL20**	N/A	deletion-duplication	17
17q21.31 (novel)	*UBTF**, HDAC5*, EFTUD2***	*RPL27*, FMNL1*, GPATCH8*, KANSL1**+, NMT1**	N/A	duplication	17
21q11.2-q22.3 (novel)	N/A	*U2AF1*+, NRIP1*, BTG3*, GABPA*, ADAMTS1*, CCT8*, BACH1, TIAM1*+, CHAF1B*, MORC3*, ETS2*+, BRWD1*+, ZBTB21*+, SIK1**+, ADARB1**+, COL6A1*+, USP25*, APP*, LTN1*, SCAF4**, PAXBP1*, SYNJ1**, ITSN1**, SLC5A3*, RUNX1+, DYRK1A**+, TTC3, ERG*+, DSCAM**+, DIP2A**+, PKNOX1*+, TRAPPC10*+, SON***	N/A	duplication	21
22q11.21	N/A	*CECR2*+, MICAL3*+, DGCR8*+, SCARF2*+, UBE2L3+, HIRA*+, MED15*+*	N/A	deletion-duplication	22
22q11.21-q11.23	N/A	*CECR2*+, MICAL3*+, DGCR8*+, SCARF2*+, UBE2L3+, MAPK1**+, SMARCB1**+, HIRA*+, MED15*+, BCR*+, KIAA1671+*	N/A	deletion	22
22q13.2-q13.33 (novel due to novel region boundaries)	*PLXNB2*+*	*PIM3*+, SHANK3**+, ST13+, RBX1*+, ZC3H7B*+, ACO2**+, TOB2+, XRCC6*+, SREBF2+, FBLN1*+, GRAMD4*+, MAPK8IP2*+, EP300**+, TCF20**+, SCUBE1*+, PHF21B*+, CELSR1*+, BRD1*+, SBF1*+*	N/A	deletion-duplication	22

*Candidate and high confidence. **NDD-risk genes according to the GeneTrek catalog. +Genes validated through our burden analysis surpassing Bonferroni correction. N/A, not applicable, no dosage sensitive genes have been found in this region.

and axon morphology, transcriptional regulation, or chromatin remodeling), so we assessed longitudinal and regional gene expression in neurotypical brains. Using RNA-seq data from Brain Span brain samples, and after a chordate standardization of the counts, we obtained a distribution of gene expression across all developmental stages in neocortex, amygdala, and cerebellum. Remarkably, we found that the top 5% most expressed genes of

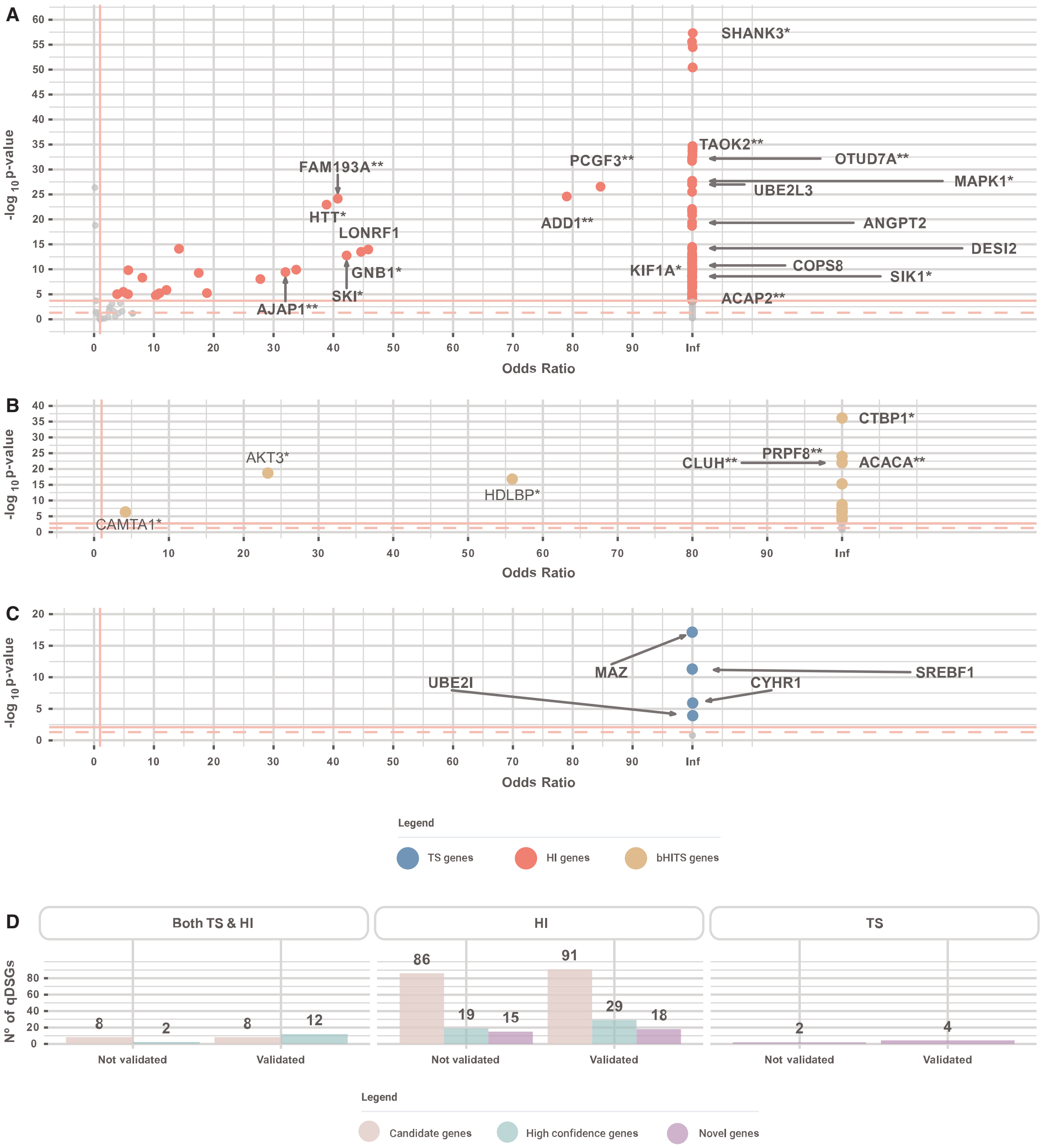

Figure 2. Validation of NDD-risk for 162 qDSGs, 22 of them novel genes never associated before with risk for NDD

Validation of qDSG in NDD-risk regions found associated to autism in affected individuals from iHART and Decipher compared with control individuals from 1000 Genomes.

(A–C) Volcano plot depicting the p values and odds ratios for qDSGs genes in NDD-risk regions: (A) HI genes, (B) bHITS genes, and (C) TS genes. Vertical red line delimits odds ratio 1, whereas horizontal red line stands for the multiple test correction threshold. Dashed red line represents $p = 0.05$. (D) Bar plot describing the number of qDSG validated according to type of novelty. *Candidate and high confidence, **NDD-risk genes according to the GeneTrek catalog.

this distribution were enriched for qDSGs including HI genes, TS genes, and bHITS genes (Figures 4, S1, and S2; Table S6). Furthermore, this enrichment could be observed throughout all stages of development up to adulthood in key brain regions such as the neocortex regions, the amygdala, and the cerebellum.

Interestingly, we observed that, among the 87 qDSGs in our list that are highly expressed in any of the tissues

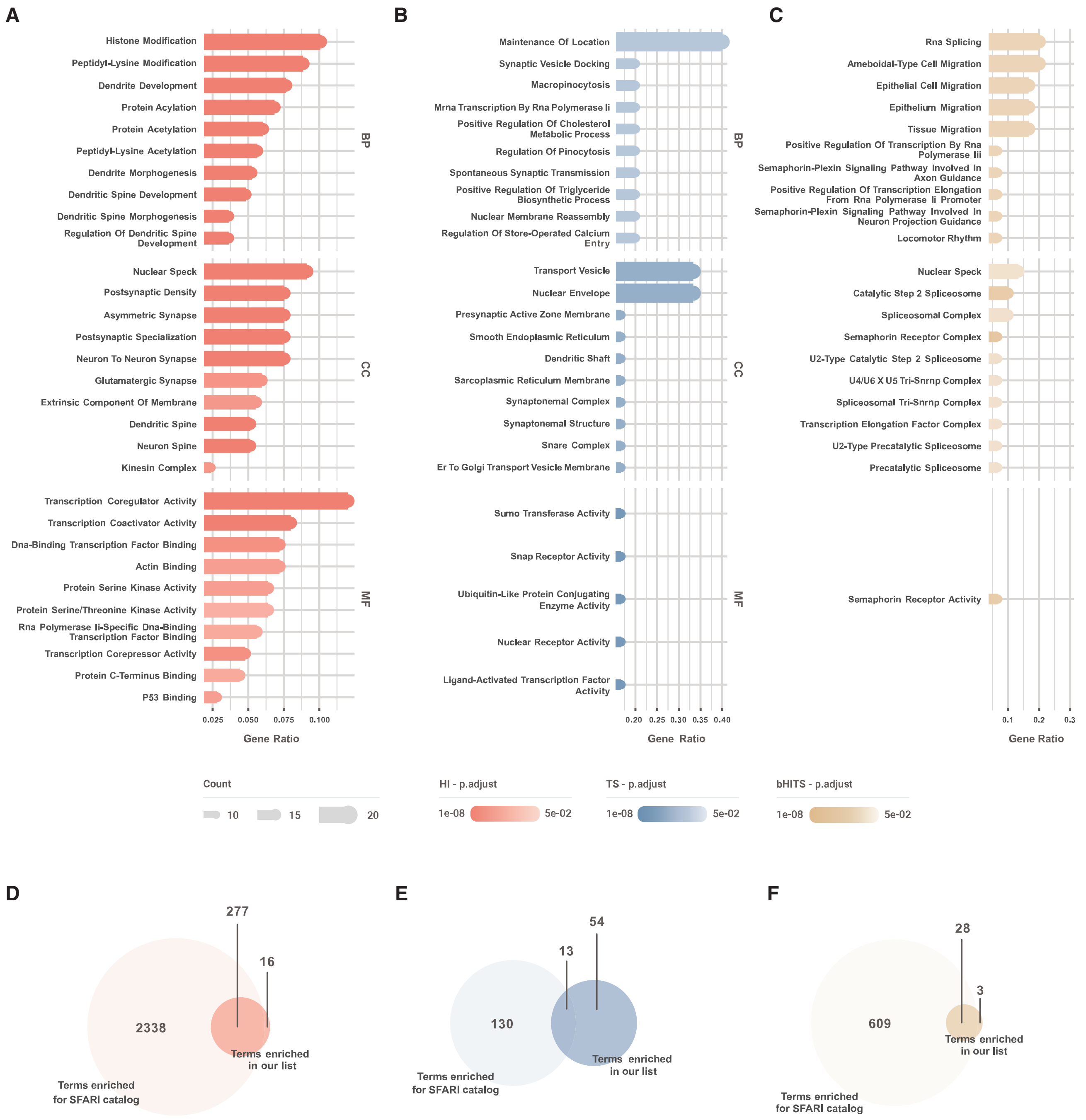

Figure 3. Pathways where qDSGs are involved, and their overlap with known NDD-risk pathways
Subset of top GO terms enriched for HI genes (A), for TS genes (B), and for bHITS genes (C). In the lower half is depicted the overlap between the GO terms enriched for our qDSGs and the GO terms enriched for the genes in the SFARI catalog of ASD-risk genes, also divided according to their dosage type, for HI genes (D), for TS genes (E), and for bHITS genes (F).

assessed (top 5% percentile), 36 of them remain in this percentile category from fetal age to adulthood, of which 8 have already been reported to be associated with autism and other NDDs (*CLIP2*, *HDLBP*, *HNRNPU*, *KIF1A*, *SBF1*, *YWHAE*, *YWHAG*, *PRKCB*; Figures S3 and S4; Table S7). In addition, these genes are related to biological processes previously associated with abnormal neurodevelopment, such as histone modification, dendrite development, synaptic transmission, muscle tissue development, semaphorin-plexin signaling pathway, or regulation of neural projection (Table S8).

qDSGs converge at PPI networks

PPI enrichment was evaluated for the 294 proteins encoded by qDSGs using STRING. Following a conservative approach for this protein interaction analysis, we created the PPI networks using only strong interaction evidence from databases and experiments. This analysis confirmed

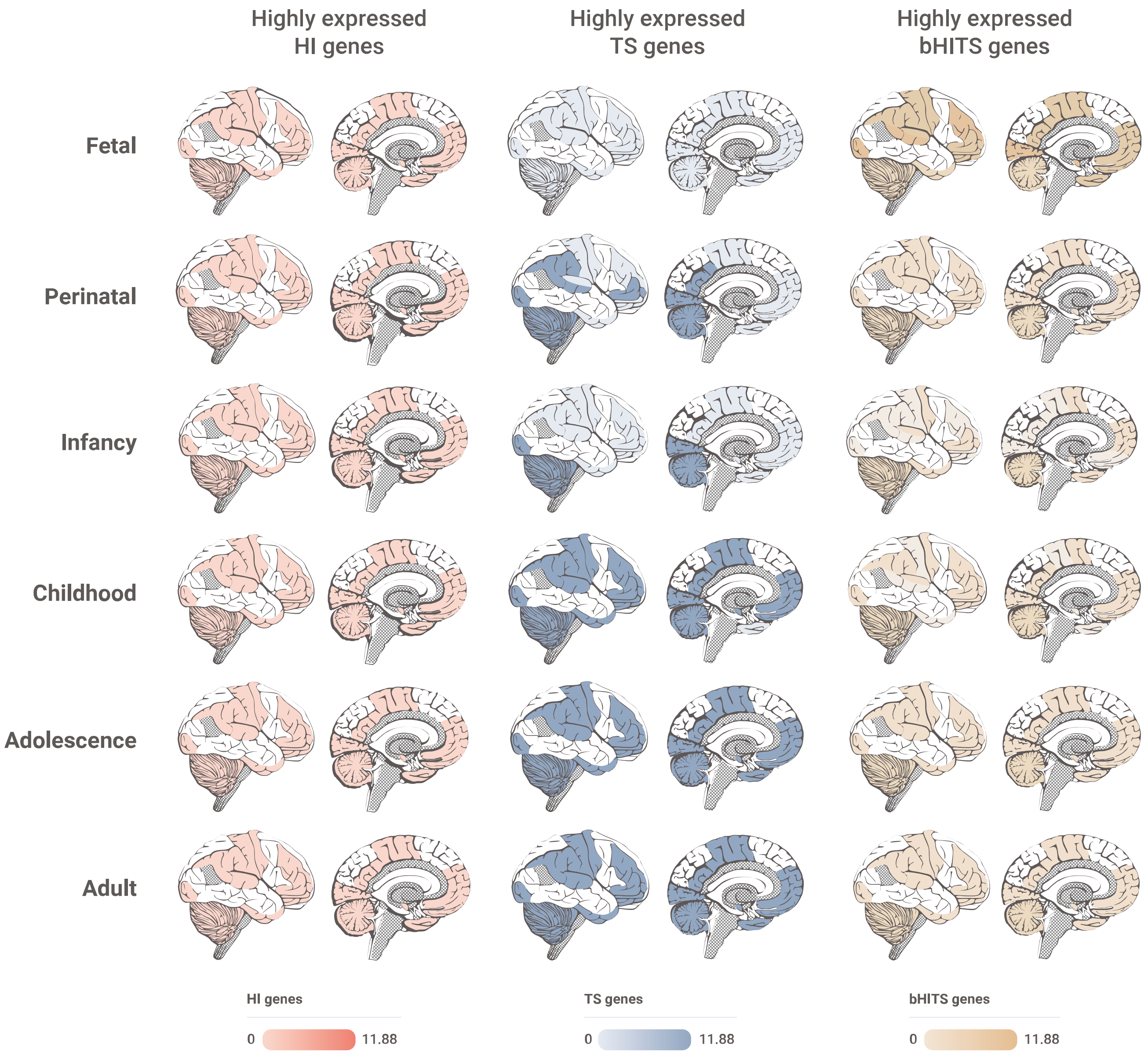

Figure 4. qDSGs are significantly highly expressed in key areas of the brain across all developmental stages
Surface and slice representation of odds ratio resulting from the enrichment analysis of qDSGs in the highly expressed ranking. Only statistically significant areas enriched with highly expressed qDSGs are colored.

a statistically significant enrichment in the interaction of the protein products of qDSGs, (expected/observed number of edges = 286/328; $p = 6.95 \times 10^{-4}$, average node degree = 2.56; cluster coefficient = 0.351; Figure 5; Table S9).

Interestingly, a high degree of direct and/or indirect interaction (distance of 1 and 2 nodes in the network, respectively) of proteins encoded by qDSGs that are located within the same NDD-risk CNV region was also found. In particular, this was observed for 13 out of the 36 (36.11%) NDD-risk CNV regions reported in this study that have at least one qDSG. For example, for the 16p13.3 region, among the 12 qDSGs identified within this region, 5 are indirectly connected and two are directly connected (Table S10). This is consistent with the fact that a higher probability of interaction is expected for genes that are close to each other on the same chromosome, due to the three-dimensional organization of the genome.[26] Noteworthy, a trend toward higher degree of protein connectivity is also observed when randomly selecting a high-confidence NDD-risk qDSG from each NDD-risk region (expected/observed number of edges = 3/6; $p = 0.08$, average node degree = 0.5; cluster coefficient = 0.333), indicating functional convergence also across NDD-risk regions reported in this study.

qDSGs are associated with the manifestation of specific clinical phenotypes

We finally investigated whether deletion or duplication of qDSGs are associated with the manifestation of specific phenotypes in carrier affected individuals. Using Decipher

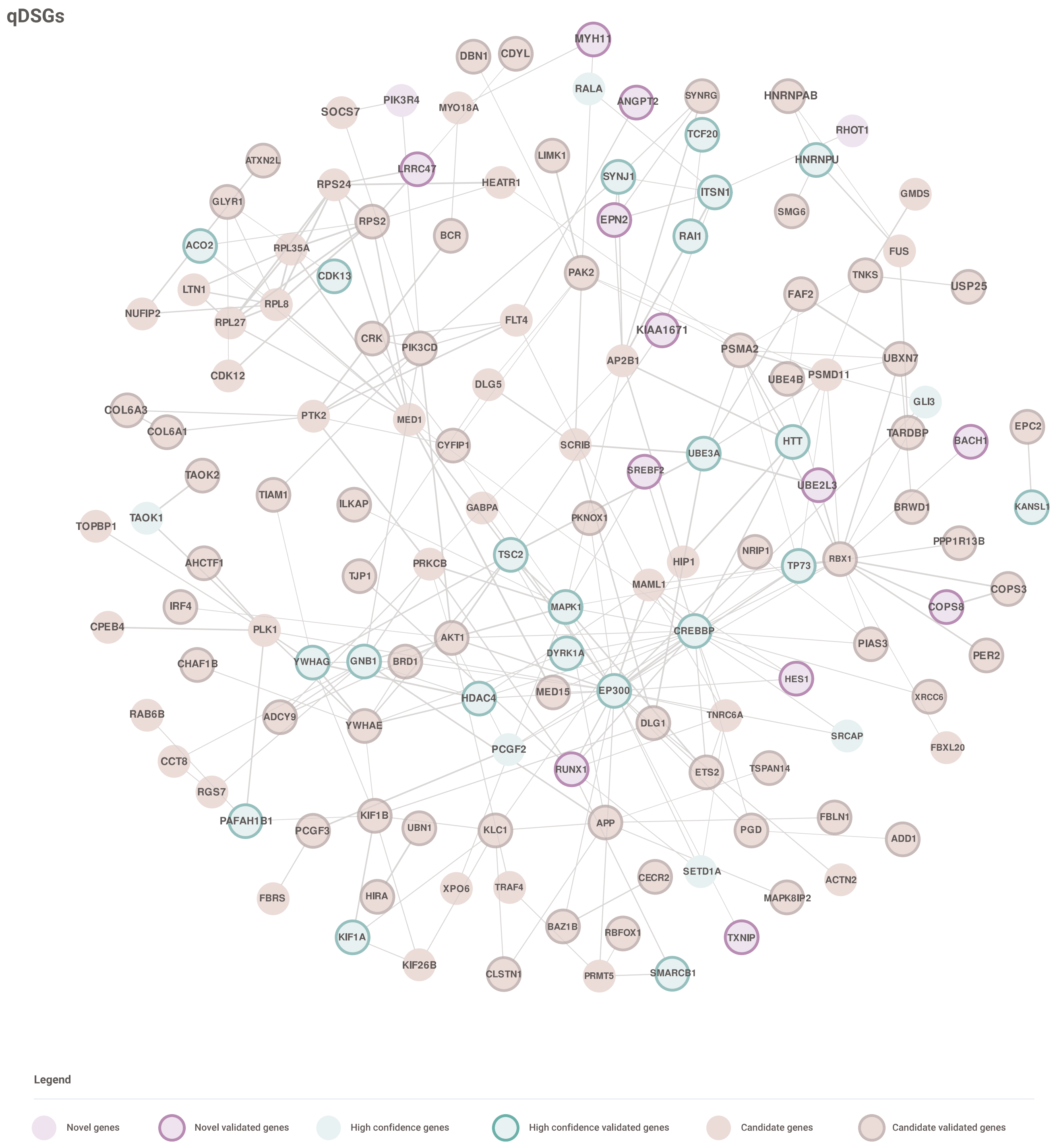

Figure 5. PPI network depicting interactions between protein products of qDSGs
Protein-protein interaction network depicting interactions of qDSGs where nodes are colored based on their evidence strength of NDD-risk. Circled nodes stand for validated NDD-risk for those genes in our analysis.

data, we found a total of 1,786 unique phenotypes for 3,708 affected individuals with deletions or duplications affecting qDSGs. Out of the 40,838 evaluated gene-phenotype relationships, a total of 1,761 were found to be statistically significant surpassing Bonferroni multiple test correction ($p < 1.22 \times 10^{-6}$). Interestingly, while 10.39% (183) of these gene-phenotype relationships can also be found reported in the Human Phenotype Ontology (HPO) database, 89.61% (1,578) of them are novel. Besides, we found that the phenotypes significantly associated with the highest number of qDSGs (Figure 6A) are common clinical signs and symptoms reported in affected individuals with NDDs, such as hypertelorism, hypoplasia of the corpus callosum, lissencephaly, or hypotonia. We also found that most of the genes with the highest number of significant gene-phenotype associations were candidate or high-confidence

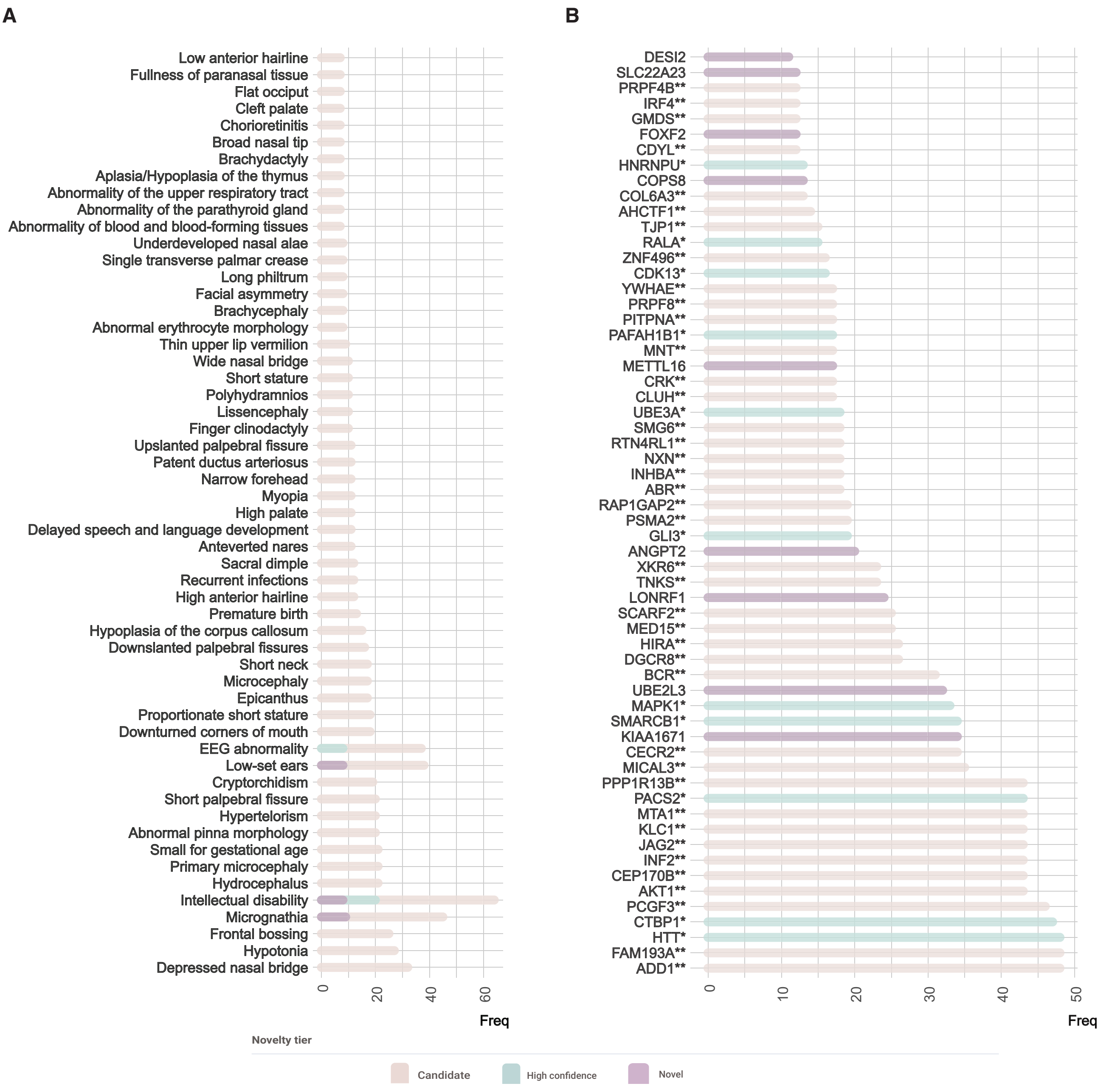

Figure 6. Phenotypes associated to most qDSGs are NDD-risk clinical signs and symptoms
(A) Histogram listing the top 60 HPO terms associated with NDD-risk genes, according to the type and number of genes they are associated to.
(B) Histogram listing the top 60 NDD-risk genes, according to the number of HPO phenotypes they are associated.

genes (Figure 6B), although some of the novel validated genes such as *COPS8* were also associated with a high number of statistically significant gene-phenotype relationships (see Table S3 for further details on the specific HPO terms associated to these genes).

Discussion

Large and rare CNVs are associated with risk for a wide range of NDDs and contribute to the shared etiology of disorders such as autism (ASD), schizophrenia (SCZ), attention deficit hyperactivity disorder (ADHD), obsessive-compulsive disorder (OCD), bipolar disorder, or intellectual disabilities (ID). By conducting a comprehensive meta-analysis of available datasets, we have validated the association of 17 previously known NDD-risk regions and found 24 novel NDD-risk regions, of which 5 partially overlap with previously known NDD-risk regions.

Interestingly, for several NDD-risk regions that we identified, deletions and/or duplications have been described in association with more than one NDD, such as ASD, ADHD,

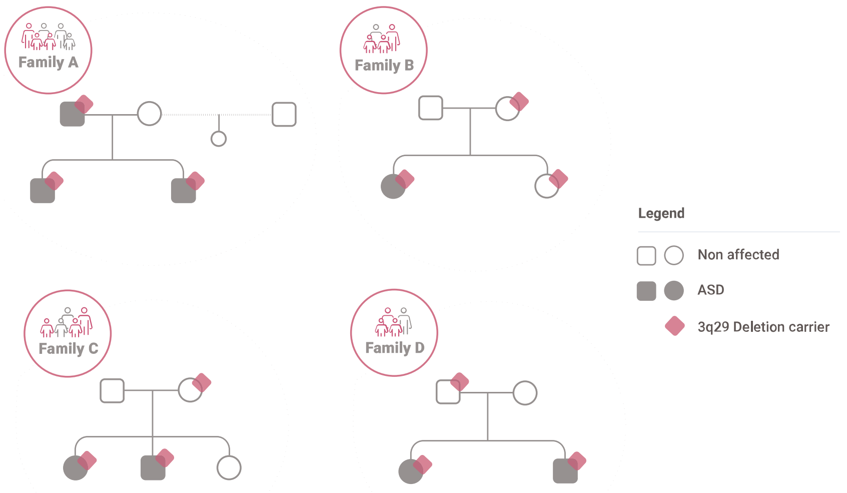

Figure 7. Pedigree for deleted CNVs on 3q29, affecting qDSG ACAP2, which was identified in four different families with a transmission rate of 1

SCZ, or ID (see supplemental information*[1]). For example, reciprocal structural variants (duplications and deletions) at region 1q21 are found at high incidence in ASD, SCZ, ADHD, OCD, ID, or epilepsy. Although there is wide phenotypical variation among affected individuals with a CNV in these region,[27,28] duplication events are more associated with autism, while high rates of deletions are shown to be more related with ID, SCZ, and epilepsy.[29] Besides, ADHD risk has been associated to affected individuals carrying both deletions and duplications, although affected individuals with a 1q21.1 deletion have higher scores for ADHD traits.[30,31] The 15q locus is another highly complex region of the human genome, being the second most common region affected by duplications in affected individuals with autism.[32] Duplications in subregions such as 15q11-q13, 15q13.2-q13.3, or 15q13.3 also show a high range of clinical heterogeneity, and beyond autism, a risk association signal is also found for affected individuals with ADHD,[33,34] SCZ,[35–38] or OCD.[39–41] This fact also highlights shared etiologies among these heterogeneous groups of disorders and supports the rationale of grouping affected individuals with NDDs for variant and gene association analysis as done in the current study.[42] Thus, even though most affected individuals in our study are diagnosed with ASD, the findings reported here are overall related with risk for abnormal brain development, which characterizes affected individuals with NDDs. Consistently, for all high-confidence NDD-risk regions identified in this study we find affected individual CNV carriers holding diagnoses of developmental delay (Table S1).

Substantial evidence indicates that the mechanism through which these disease phenotypes are triggered is the dosage sensitivity of genes affected by CNV. These CNVs are known to affect broad regions of the genome, and pinpointing which genes are responsible for causing the phenotypic profile remains a challenge. Through our results, we confirmed previous evidence that indicates that dosage variation is a key mechanism leading to this type of disorder, since NDD-risk regions, both novel and previously reported, were significantly enriched for dosage-sensitive genes previously associated with risk for ASD and NDDs.

In addition, we observed that deletion variants affecting HI genes enclosed in the NDD-associated regions are more transmitted to offspring with an ASD diagnosis than expected by chance. Furthermore, HI and/or TS genes within NDD-risk regions (qDSGs) were found to be significantly involved in biological process or functions disrupted in affected individuals with NDDs, such as synapsis, dendrite morphology, axon guidance, histone/RNA biding or semaphorin pathway, among others. Many of these pathways are essential in the central nervous system throughout an individual's entire life span. Linked to this fact, we found a significant overrepresentation of these dosage-sensitive genes in the top 5% percentile of more expressed genes across all stages of development in brain tissue. In particular, we found that 36 qDSGs are highly expressed in the brain from the fetal stage until adulthood, and they are involved in regulation of core biological pathways, such as mitochondrion organization, histone modification, selective molecular interaction, or RNA splicing, as well as in specific nervous system processes, such as neuromuscular control, dendrite development, or modulation of synaptic transmission. These findings provide evidence that mechanisms of gene dosage dysfunction during brain development can persist in adult individuals, which suggests that some targeted pharmacological therapies might still be beneficial in adulthood.

In addition, in independent cohorts to those used for the meta-analysis, gene-NDD-risk association was validated for 162 out of 294 qDSGs, including 22 novel NDD-risk genes. Importantly, multiple NDD-risk genes were identified and/or validated per CNV for most of the NDD-risk CNVs identified in this study, which provides additional evidence for the pathophysiology of NDDs caused by large and rare CNVs not necessarily fitting into a monogenic model. This complex interaction model where candidate genes within each region interact with each other to influence a broad clinical profile is also ratified by the high connectivity we have observed in the PPI network, which also applies for genes within the same risk regions. Such a multigenic pathogenicity model is consistent with a complex genetic architecture of NDDs, in which hundreds of genes with varying size effects and complex interactions exert an impact on developmental traits.

Among the 22 novel NDD-risk genes identified in this study, there is the HI gene *COPS8*, located in novel risk region 2q37.3. This gene is involved in both MAPK cascade regulation and histone modification, and it interacts in the described PPI network with genes previously reported in association with autism, such as *COPS3*, *RBX1*, and *PSMD11*. Deletions in the 2q37.3 region have been previously reported in affected individuals with autism[43] and other NDDs[44,45] but *COPS8* had never been associated as a critical gene for this region. Another identified novel NDD-risk gene is *DESI2*, located in the previously known NDD-risk region 1q43-q44. *DESI2* regulates the modification of proteins by small ubiquitin-like modifier which is an essential posttranslational regulatory mechanism whose defects been proven to have an important influence in axonal trafficking, neural plasticity, brain development, and neurodegeneration.[46] We found several NDD-related clinical signs and symptoms as significantly associated to this gene, such as EEG abnormality, epicanthus, micrognathia, prominent metopic ridge, microcephaly, or hypoplasia of the corpus callosum (Table S3).

On the other hand, we also validated candidate genes that had not been established yet as high-confidence NDD-risk genes. For instance, we confirmed *ACAP2* as a high-confidence NDD-risk gene located in the 3q29 region, which was previously well known to be associated with risk for different NDDs, but for which the driver gene had not been yet identified. We found deletions of this gene to be transmitted to all the children with ASD in four multiplex families from iHART (Figure 7). Animal knockout models have shown that *ACAP2* is required to promote tunneling nanotubes and vesicle trafficking,[47] cytoskeletal structures that have been shown to be essential in neuronal morphology, including axon growth and guidance, dendrite development and plasticity, and synapse formation (see supplemental information*[2]).

This study also provides additional association evidence for genes that have been extensively reported in the literature as high-confidence NDD-risk genes, such as *MTOR*. Pharmacological inhibition of mTOR has been described as effective in rescuing synaptic surplus and functional hyperconnectivity observed in some affected individuals with autism.[48] *MTOR* is known to be involved in reward/aversion processing as well as in positive/negative reinforcement learning, both circuits being disrupted in neuropsychiatric disorders (SCZ, ADHD, ASD, Tourette syndrome, conduct disorder/oppositional defiant disorder, Fragile X syndrome, Prader-Willi syndrome, Williams syndrome, Angelman syndrome, and Rett syndrome).[49,50]

By conducting validation of the findings in independent cohorts and through multiple gene set enrichment analyses we have mitigated limitations of the current study, including: (1) scarcity of information about potential confounding factors such as individual gender and ancestry and (2) resolution of the array platforms used to ascertain the CNVs. Overall, the research findings described in this study represent a step forward to increase genetic diagnostic yield in NDDs, enable the characterization of new NDD syndromes, and to expand the knowledge bases on molecular processes implicated in these conditions.

Web resources

Brain Span: www.brainspan.org/rnaseq

GeneTrek: https://genetrek.pasteur.fr

SFARI: https://gene.sfari.org

STRING: https://www.string-db.org

Supplemental information

Supplemental information can be found online at https://doi.org/10.1016/j.xhgg.2024.100316.

Acknowledgments

This study makes use of data generated by the DECIPHER community. A full list of centers who contributed to the generation of the data is available from https://deciphergenomics.org/about/stats and via email from contact@deciphergenomics.org. DECIPHER is hosted by EMBL-EBI and funding for the DECIPHER project was provided by the Wellcome Trust (grant no. WT223718/Z/21/Z). Access to the individual CNV calls from the iHart cohort[17] was granted by the board of trustees of the Leland Stanford Junior University. We acknowledge the Hartwell Foundation for supporting whole-genome sequencing and the creation of the iHART database, as well as the Simons Foundation who provided additional support for the genome sequencing. We appreciate obtaining access to genetic data on SFARI Base (https:/base.SFARI.org) and the Database of Genomic Variants (http://dgv.tcag.ca/dgv/app/home). S.A.C. was supported by industrial doctorate grant 2021DI056 from the Agència de Gestió d'Ajuts Universitaris i de Recerca (AGAUR) of the Generalitat de Catalunya.

Author contributions

S.A. was primarily responsible for conducting the experiments, analyzing the data, and writing the initial draft of the manuscript. X.G. contributed to the study desing, data processing and assisted in revising the manuscript. L.D. was involved in the experimental design and provided critical revisions to the manuscript. M.C. contributed to the project conceptualisation, supervised the study, and assisted in the interpretation of data. E.G. contributed to data analysis, and helped in the drafting and revision of the manuscript. L.P.C. led the overall project, was responsible for the study design and supervision, and critically reviewed and revised the manuscript.

Declaration of interests

S.A.C., X.G., L.P.-C., E.G., and L.D. are STALICLA employees, a company advancing treatment candidates for subgroups of affected individuals with NDDs.

Received: February 2, 2024
Accepted: June 3, 2024

References

1. Itsara, A., Wu, H., Smith, J.D., Nickerson, D.A., Romieu, I., London, S.J., and Eichler, E.E. (2010). De novo rates and selection of large copy number variation. Genome Res. *20*, 1469–1481.
2. Pos, O., Radvanszky, J., Buglyo, G., Pos, Z., Rusnakova, D., Nagy, B., and Szemes, T. (2021). DNA copy number variation: Main characteristics, evolutionary significance, and pathological aspects. Biomed. J. *44*, 548–559.
3. Bacchelli, E., Cameli, C., Viggiano, M., Igliozzi, R., Mancini, A., Tancredi, R., Battaglia, A., and Maestrini, E. (2020). An integrated analysis of rare CNV and exome variation in Autism Spectrum Disorder using the Infinium PsychArray. Sci. Rep. *10*, 3198.
4. Shishido, E., Aleksic, B., and Ozaki, N. (2014). Copy-number variation in the pathogenesis of autism spectrum disorder. Psychiatry Clin. Neurosci. *68*, 85–95.
5. Barone, J., Smith, M., Kendall, K., Owen, M., O'Donovan, M., and Kirov, G. (2019). The rate of *de novo* CNVs in healthy controls.
6. Girirajan, S., Brkanac, Z., Coe, B.P., Baker, C., Vives, L., Vu, T.H., Shafer, N., Bernier, R., Ferrero, G.B., Silengo, M., et al. (2011). Relative burden of large CNVs on a range of neurodevelopmental phenotypes. PLoS Genet. *7*, e1002334.
7. Nishino, J., Ochi, H., Kochi, Y., Tsunoda, T., and Matsui, S. (2018). Sample Size for Successful Genome-Wide Association Study of Major Depressive Disorder. Front. Genet. *9*, 227.
8. Collins, R.L., Glessner, J.T., Porcu, E., Lepamets, M., Brandon, R., Lauricella, C., Han, L., Morley, T., Niestroj, L.M., Ulirsch, J., et al. (2022). A cross-disorder dosage sensitivity map of the human genome. Cell *185*, 3041–3055.e25.
9. Marshall, C.R., Howrigan, D.P., Merico, D., Thiruvahindrapuram, B., Wu, W., Greer, D.S., Antaki, D., Shetty, A., Holmans, P.A., Pinto, D., et al. (2017). Contribution of copy number variants to schizophrenia from a genome-wide study of 41,321 subjects. Nat. Genet. *49*, 27–35.
10. Sanders, S.J., He, X., Willsey, A.J., Ercan-Sencicek, A.G., Samocha, K.E., Cicek, A.E., Murtha, M.T., Bal, V.H., Bishop, S.L., Dong, S., et al. (2015). Insights into Autism Spectrum Disorder Genomic Architecture and Biology from 71 Risk Loci. Neuron *87*, 1215–1233.
11. Zhang, F., Gu, W., Hurles, M.E., and Lupski, J.R. (2009). Copy number variation in human health, disease, and evolution. Annu. Rev. Genomics Hum. Genet. *10*, 451–481.
12. Rice, A.M., and McLysaght, A. (2017). Dosage-sensitive genes in evolution and disease. BMC Biol. *15*, 78.
13. Leblond, C.S., Le, T.L., Malesys, S., Cliquet, F., Tabet, A.C., Delorme, R., Rolland, T., and Bourgeron, T. (2021). Operative list of genes associated with autism and neurodevelopmental disorders based on database review. Mol. Cell. Neurosci. *113*, 103623.
14. Raudvere, U., Kolberg, L., Kuzmin, I., Arak, T., Adler, P., Peterson, H., and Vilo, J. (2019). g:Profiler: a web server for functional enrichment analysis and conversions of gene lists (2019 update). Nucleic Acids Res. *47*, W191–W198.
15. Ashburner, M., Ball, C.A., Blake, J.A., Botstein, D., Butler, H., Cherry, J.M., Davis, A.P., Dolinski, K., Dwight, S.S., Eppig, J.T., et al. (2000). Gene ontology: tool for the unification of biology. The Gene Ontology Consortium. Nat. Genet. *25*, 25–29.
16. Gene Ontology Consortium (2021). The Gene Ontology resource: enriching a GOld mine. Nucleic Acids Res. *49*, D325–D334.
17. Ruzzo, E.K., Perez-Cano, L., Jung, J.Y., Wang, L.K., Kashef-Haghighi, D., Hartl, C., Singh, C., Xu, J., Hoekstra, J.N., Leventhal, O., et al. (2019). Inherited and De Novo Genetic Risk for Autism Impacts Shared Networks. Cell *178*, 850–866.e26.
18. Firth, H.V., Richards, S.M., Bevan, A.P., Clayton, S., Corpas, M., Rajan, D., Van Vooren, S., Moreau, Y., Pettett, R.M., and Carter, N.P. (2009). DECIPHER: Database of Chromosomal Imbalance and Phenotype in Humans Using Ensembl Resources. Am. J. Hum. Genet. *84*, 524–533.
19. MacDonald, J.R., Ziman, R., Yuen, R.K.C., Feuk, L., and Scherer, S.W. (2014). The Database of Genomic Variants: a curated collection of structural variation in the human genome. Nucleic Acids Res. *42*, D986–D992.
20. Karczewski, K.J., Francioli, L.C., Tiao, G., Cummings, B.B., Alföldi, J., Wang, Q., Collins, R.L., Laricchia, K.M., Ganna, A., Birnbaum, D.P., et al. (2020). The mutational constraint spectrum quantified from variation in 141,456 humans. Nature *581*, 434–443.
21. Anders, S., and Huber, W. (2010). Differential expression analysis for sequence count data. Genome Biol. *11*, R106.
22. Satterstrom, F.K., Kosmicki, J.A., Wang, J., Breen, M.S., De Rubeis, S., An, J.Y., Peng, M., Collins, R., Grove, J., Klei, L., et al. (2020). Large-Scale Exome Sequencing Study Implicates Both Developmental and Functional Changes in the Neurobiology of Autism. Cell *180*, 568–584.e23.
23. Trost, B., Thiruvahindrapuram, B., Chan, A.J.S., Engchuan, W., Higginbotham, E.J., Howe, J.L., Loureiro, L.O., Reuter, M.S., Roshandel, D., Whitney, J., et al. (2022). Genomic architecture of autism from comprehensive whole-genome sequence annotation. Cell *185*, 4409–4427.e18.
24. D'Souza, L., Channakkar, A.S., and Muralidharan, B. (2021). Chromatin remodelling complexes in cerebral cortex development and neurodevelopmental disorders. Neurochem. Int. *147*, 105055.
25. Sokpor, G., Castro-Hernandez, R., Rosenbusch, J., Staiger, J.F., and Tuoc, T. (2018). ATP-Dependent Chromatin Remodeling During Cortical Neurogenesis. Front. Neurosci. *12*, 226.
26. Dandekar, T., Snel, B., Huynen, M., and Bork, P. (1998). Conservation of gene order: a fingerprint of proteins that physically interact. Trends Biochem. Sci. *23*, 324–328.
27. Grunblatt, E., Oneda, B., Ekici, A.B., Ball, J., Geissler, J., Uebe, S., Romanos, M., Rauch, A., and Walitza, S. (2017). High resolution chromosomal microarray analysis in paediatric obsessive-compulsive disorder. BMC Med. Genom. *10*, 68.
28. Mefford, H.C., Sharp, A.J., Baker, C., Itsara, A., Jiang, Z., Buysse, K., Huang, S., Maloney, V.K., Crolla, J.A., Baralle, D., et al. (2008). Recurrent rearrangements of chromosome 1q21.1 and variable pediatric phenotypes. N. Engl. J. Med. *359*, 1685–1699.
29. Coe, B.P., Girirajan, S., and Eichler, E.E. (2012). The genetic variability and commonality of neurodevelopmental disease. Am. J. Med. Genet. C Semin. Med. Genet. *160C*, 118–129.
30. Gudmundsson, O.O., Walters, G.B., Ingason, A., Johansson, S., Zayats, T., Athanasiu, L., Sonderby, I.E., Gustafsson, O., Nawaz, M.S., Jonsson, G.F., et al. (2019). Attention-deficit hyperactivity disorder shares copy number variant risk with schizophrenia and autism spectrum disorder. Transl. Psychiatry *9*, 258.
31. Linden, S.C., Watson, C.J., Smith, J., Chawner, S.J.R.A., Lancaster, T.M., Evans, F., Williams, N., Skuse, D., Raymond, F.L., Hall, J., et al. (2021). The psychiatric phenotypes of 1q21 distal deletion and duplication. Transl. Psychiatry *11*, 105.

32. Depienne, C., Moreno-De-Luca, D., Heron, D., Bouteiller, D., Gennetier, A., Delorme, R., Chaste, P., Siffroi, J.P., Chantot-Bastaraud, S., Benyahia, B., et al. (2009). Screening for genomic rearrangements and methylation abnormalities of the 15q11-q13 region in autism spectrum disorders. Biol. Psychiatry *66*, 349–359.
33. Thomas, J.A., Johnson, J., Peterson Kraai, T.L., Wilson, R., Tartaglia, N., LeRoux, J., Beischel, L., McGavran, L., and Hagerman, R.J. (2003). Genetic and clinical characterization of patients with an interstitial duplication 15q11-q13, emphasizing behavioral phenotype and response to treatment. Am. J. Med. Genet. *119A*, 111–120.
34. Williams, N.M., Franke, B., Mick, E., Anney, R.J.L., Freitag, C.M., Gill, M., Thapar, A., O'Donovan, M.C., Owen, M.J., Holmans, P., et al. (2012). Genome-wide analysis of copy number variants in attention deficit hyperactivity disorder: the role of rare variants and duplications at 15q13.3. Am. J. Psychiatry *169*, 195–204.
35. Bassett, A.S. (2011). Parental origin, DNA structure, and the schizophrenia spectrum. Am. J. Psychiatry *168*, 350–353.
36. Costain, G., Lionel, A.C., Merico, D., Forsythe, P., Russell, K., Lowther, C., Yuen, T., Husted, J., Stavropoulos, D.J., Speevak, M., et al. (2013). Pathogenic rare copy number variants in community-based schizophrenia suggest a potential role for clinical microarrays. Hum. Mol. Genet. *22*, 4485–4501.
37. Ingason, A., Kirov, G., Giegling, I., Hansen, T., Isles, A.R., Jakobsen, K.D., Kristinsson, K.T., le Roux, L., Gustafsson, O., Craddock, N., et al. (2011). Maternally derived microduplications at 15q11-q13: implication of imprinted genes in psychotic illness. Am. J. Psychiatry *168*, 408–417.
38. Rees, E., Walters, J.T.R., Georgieva, L., Isles, A.R., Chambert, K.D., Richards, A.L., Mahoney-Davies, G., Legge, S.E., Moran, J.L., McCarroll, S.A., et al. (2014). Analysis of copy number variations at 15 schizophrenia-associated loci. Br. J. Psychiatry *204*, 108–114.
39. Cox, D.M., and Butler, M.G. (2015). The 15q11.2 BP1-BP2 microdeletion syndrome: a review. Int. J. Mol. Sci. *16*, 4068–4082.
40. Vanlerberghe, C., Petit, F., Malan, V., Vincent-Delorme, C., Bouquillon, S., Boute, O., Holder-Espinasse, M., Delobel, B., Duban, B., Vallee, L., et al. (2015). 15q11.2 microdeletion (BP1-BP2) and developmental delay, behaviour issues, epilepsy and congenital heart disease: a series of 52 patients. Eur. J. Med. Genet. *58*, 140–147.
41. Zarrei, M., Burton, C.L., Engchuan, W., Young, E.J., Higginbotham, E.J., MacDonald, J.R., Trost, B., Chan, A.J.S., Walker, S., Lamoureux, S., et al. (2019). A large data resource of genomic copy number variation across neurodevelopmental disorders. NPJ Genom. Med. *4*, 26.
42. Gandal, M.J., Haney, J.R., Parikshak, N.N., Leppa, V., Ramaswami, G., Hartl, C., Schork, A.J., Appadurai, V., Buil, A., Werge, T.M., et al. (2019). Shared Molecular Neuropathology Across Major Psychiatric Disorders Parallels Polygenic Overlap. Focus *17*, 66–72.
43. Wassink, T.H., Piven, J., Vieland, V.J., Jenkins, L., Frantz, R., Bartlett, C.W., Goedken, R., Childress, D., Spence, M.A., Smith, M., and Sheffield, V.C. (2005). Evaluation of the chromosome 2q37.3 gene CENTG2 as an autism susceptibility gene. Am. J. Med. Genet. B Neuropsychiatr. Genet. *136B*, 36–44.
44. Aldred, M.A., Sanford, R.O.C., Thomas, N.S., Barrow, M.A., Wilson, L.C., Brueton, L.A., Bonaglia, M.C., Hennekam, R.C.M., Eng, C., Dennis, N.R., and Trembath, R.C. (2004). Molecular analysis of 20 patients with 2q37.3 monosomy: definition of minimum deletion intervals for key phenotypes. J. Med. Genet. *41*, 433–439.
45. Ogura, K., Takeshita, K., Arakawa, C., Shimojima, K., and Yamamoto, T. (2014). Neuropsychological profiles of patients with 2q37.3 deletion associated with developmental dyspraxia. Am. J. Med. Genet. B Neuropsychiatr. Genet. *165B*, 684–690.
46. Droescher, M., Chaugule, V.K., and Pichler, A. (2013). SUMO rules: regulatory concepts and their implication in neurologic functions. NeuroMolecular Med. *15*, 639–660.
47. Bhat, S., Ljubojevic, N., Zhu, S., Fukuda, M., Echard, A., and Zurzolo, C. (2020). Rab35 and its effectors promote formation of tunneling nanotubes in neuronal cells. Sci. Rep. *10*, 16803.
48. Pagani, M., Barsotti, N., Bertero, A., Trakoshis, S., Ulysse, L., Locarno, A., Miseviciute, I., De Felice, A., Canella, C., Supekar, K., et al. (2021). mTOR-related synaptic pathology causes autism spectrum disorder-associated functional hyperconnectivity. Nat. Commun. *12*, 6084.
49. Bryant, C.D., and Yazdani, N. (2016). RNA-binding proteins, neural development and the addictions. Genes Brain Behav. *15*, 169–186.
50. Qiu, T., Ruan, M.A.R., Lynch, W.B., Cox, J.W., Beierle, J.A., Yao, E.J., Kandola, A., Chen, M.M., Kelliher, J.C., Babbs, R.K., et al. (2021). The methamphetamine-induced RNA targetome of hnRNP H in Hnrnph1 mutants showing reduced dopamine release and behavior. Preprint at bioRxiv. https://doi.org/10.1101/2021.07.06.451358.

HGGA, Volume *5*

Supplemental information

Identification of novel driver risk genes in CNV loci associated with neurodevelopmental disorders

Sara Azidane, Xavier Gallego, Lynn Durham, Mario Cáceres, Emre Guney, and Laura Pérez-Cano

Supplemental information:

Table of Contents

Supplemental figures: *2*

Supplemental Figure 1. Density plot showing the distribution of normalized RNA-seq counts across the different developmental stages. 2

Supplemental Figure 2. Number of highly expressed genes in different brain regions across all human development. 3

Supplemental Figure 3. 36 qDSGs are highly expressed in key areas of the brain across all developmental stages. 4

Supplemental Figure 4. Bar plot depicting Odds Ratios from significantly highly expressed qDSGs in key areas of the brain across all developmental stages. 5

Supplemental Methods: *6*

Meta-analysis methodology 6

Standardization procedure 6

Patient Identifiers standardization 7

Supplemental Information: *8*

Shared etiology of NDDs [*1] 8

Details about other validated NDD-risk genes [*2] 8

Supplemental references: *9*

Supplemental figures:

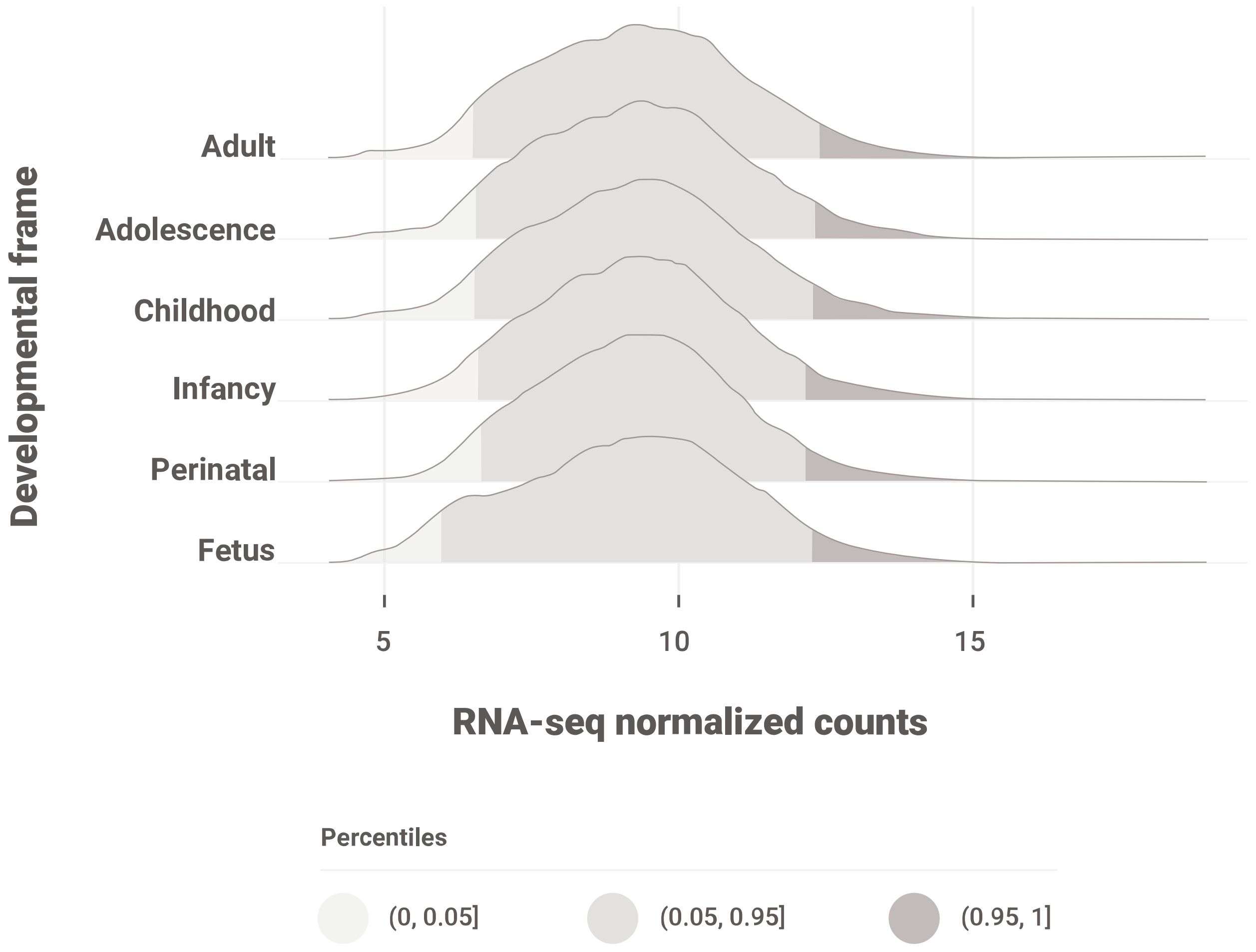

Supplemental Figure 1. Density plot showing the distribution of normalized RNA-seq counts across the different developmental stages.

In different colors are shown the perceptual ranges used to classify high expression (yellow, 95% percentile), medium expression (grey, 5% to 95%) and low expression (blue, 5% percentile) genes.

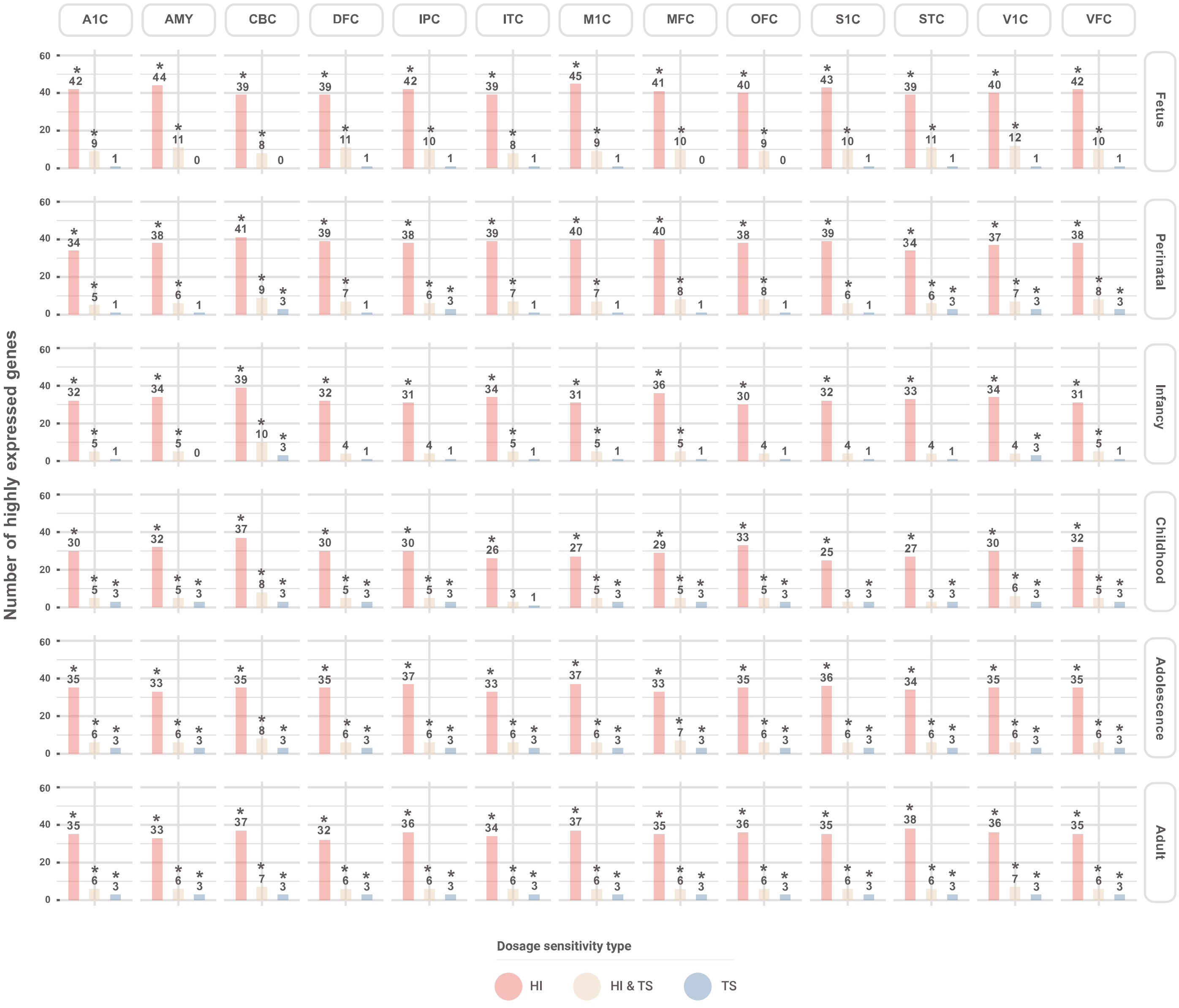

Supplemental Figure 2. Number of highly expressed genes in different brain regions across all human development.

Bar plot showing the number of highly expressed genes, i.e., with a range of expression in the top 5% percentile, in each brain region at different stages of development. The asterisk accompanying the bars indicates that this number of genes is significantly representative, i.e., the most highly expressed genes in that brain tissue at that developmental stage are enriched with our qDSGs.

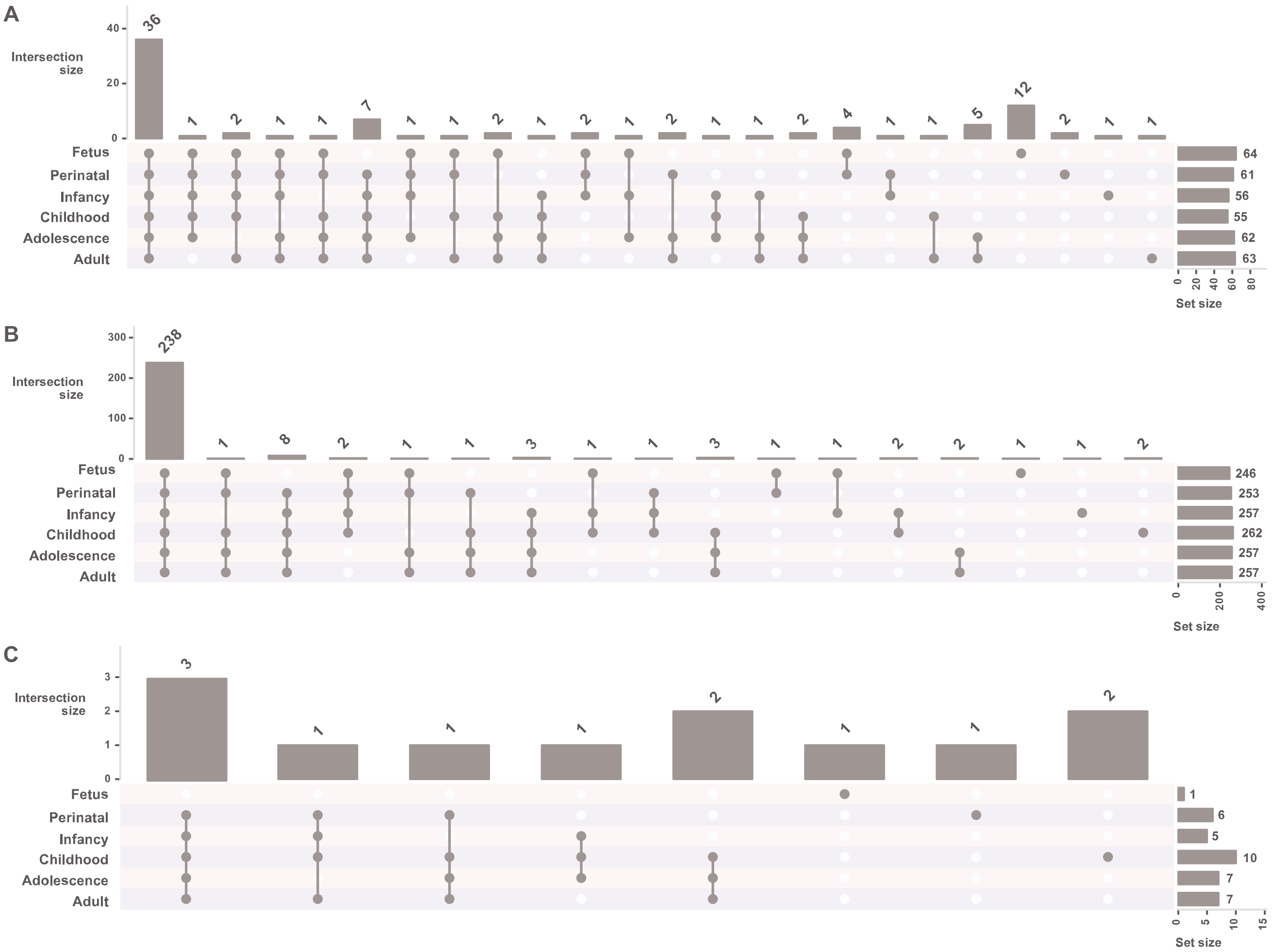

Supplemental Figure 3. 36 qDSGs are highly expressed in key areas of the brain across all developmental stages.

Venn diagram on the number of genes in each expression rank, i.e. high (A), medium (B) or low (C) expressed.

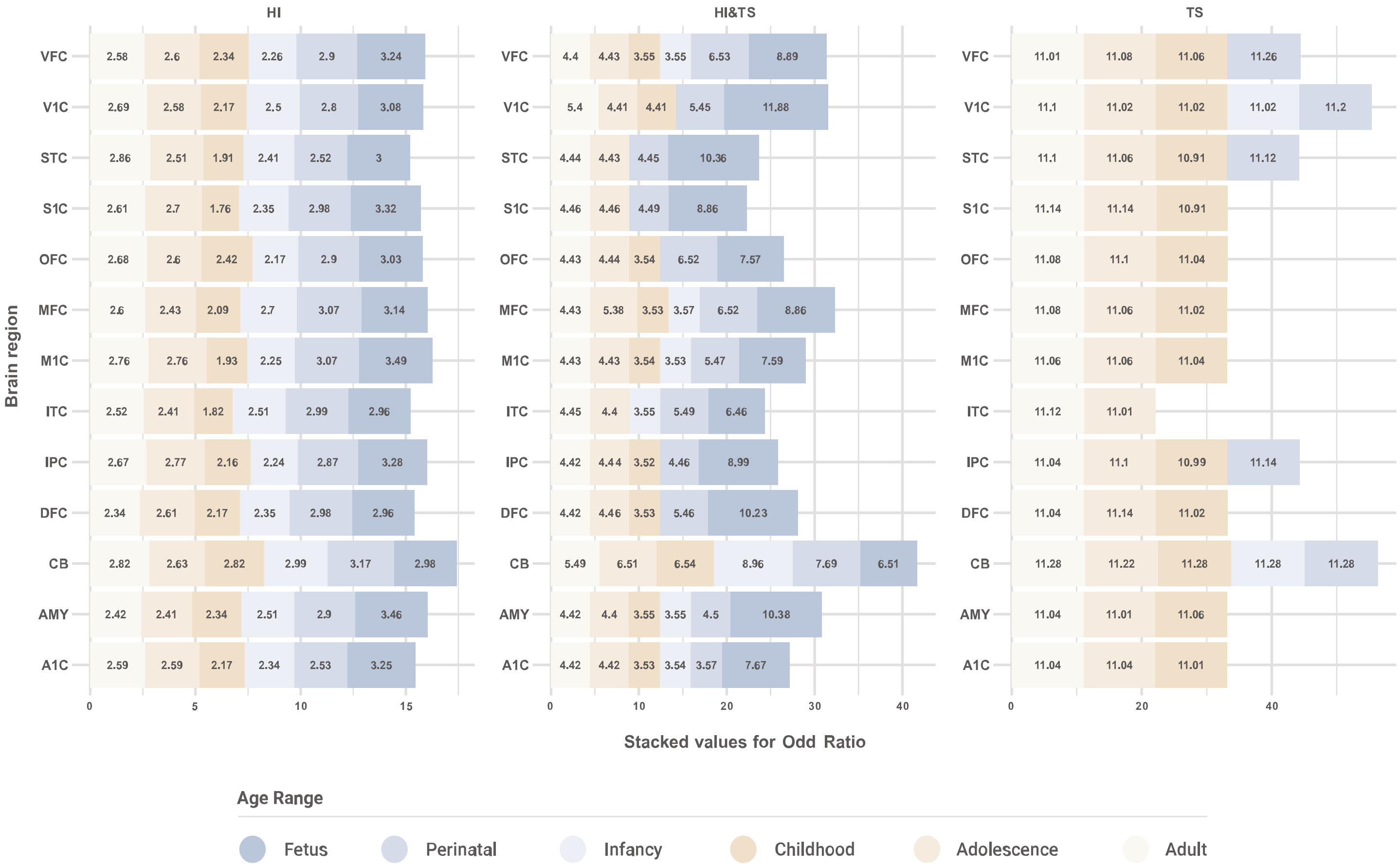

Supplemental Figure 4. Bar plot depicting Odds Ratios from significantly highly expressed qDSGs in key areas of the brain across all developmental stages.

Odd Ratios for qDSGs in each expression rank, i.e. high (A), medium (B) or low (C) expressed.

Supplemental Methods:

Meta-analysis methodology

The Gene SFARI Copy Number Variants (CNVs) database (https://gene.SFARI.org/database/cnv/) entails a collection of annotated genomic regions that includes information such as number of reported patients with NDDs and controls carrying deletions and/or duplications, ASD associated genes within these regions, genomic coordinates or detection platforms. Currently, there are two versions of this database: an archive version, more extensive, which includes CNVs reported throughout the entire genome and which do not necessarily have a significant relevance in autism, and an updated version, which only includes those regions that are highly significant in the autistic phenotype.

Data contained in SFARI website was extracted using a customized web scraper. Through this procedure, we obtained information of cases and controls, at the single patient level as well as the characteristics of the cohorts in which these probands were included.

Supplemental information table 1. Overview of data obtained from SFARI collection that were used in the statistical meta-analysis.

**Cases:** copy number variant carriers with an NDD diagnosis	Individual dataset	• Total initial number of carriers included in SFARI before the standardization: 23.907<br>• Total number of carriers after standardization procedure: 16.999<br>• Number of carriers included in our statistical analysis, after assessing cohort overlap and removing duplicates: 11,614
	Population dataset	• Initial number of studies included in SFARI: 565<br>• Studies included in the statistical test: 14<br>• Total population: 61,817
**Control:** copy number variant carriers without an NDD diagonisis.	Individual dataset	• Total initial number of carriers included in SFARI before the standardization: 6,949<br>• Total number of carriers after post-standardization procedure: 5,498<br>• Number of control carriers included in our statistical analysis, after assessing cohort overlap and removing duplicates: 4,031
	Population dataset	• Initial number of control studies included in SFARI: 105<br>• Studies included in the statistical test: 10<br>• Total population: 52,126

Standardization procedure

Bringing together formats and characteristics of different data fields under a common framework is a critical process in the study of large-scale cohorts. In particular, medical data can involve a high level of heterogeneity, due to the intrinsic complexity of biological processes and the disparity between techniques, years of study or even the healthcare facilities where analyses have been undertaken. Once

the SFARI data was obtained we detected several issues for downstream data processing (e.g., duplicate probands, inconsistencies in genomic coordinates reported or positions reported in different builds). To address data quality issues, we then proceeded to implement a data processing and standardization step, as detailed below divided by category of data intervention.

Patient Identifiers standardization

Individual patient identifiers standardization was crucial to carry out a reliable statistical analysis, since SFARI database includes duplicate entries both in cases and control samples. This is because numerous studies that were performed for the same region, based on the same case/control cohorts, are stored in SFARI database as separate entries. This fact is captured in the significant reduction that results, especially in cases, between the number of carriers before and after the standardization of the individual identifiers. (Supplemental table 1).

To resolve duplicates, we evaluated individually each of the papers of the studies from which the patients were obtained. Due to the high number of studies included, 565 case studies and 106 control studies, a first filtering was made selecting only those with more than 100 probands. This returned a total of 34 case studies and 13 control studies. We then reviewed all of them to identify the cohort used in their studies. To avoid overlapping cohorts, we selected those that did not share any patients among them either based on geographic location or because they came from different consortia.

Supplemental information table 2. Cohorts employed in this meta-analysis.

Study	PMID	Case Cohort Origin	Control Cohort Origin
**Yin CL , et al. (2016)**	27042285	Han Chinese	Han Chinese Cell and Genome Bank (HCCGB)
**Engchuan W , et al. (2015)**	25783485	AGP	SAGE (Study of Addiction Genetics and Environment), Ontario Colorectal Cancer study, HABC (Health Aging and Body Composition)
**Kaminsky EB , et al. (2011)**	21844811	ISCA	Portugal, UK and Sweden controls, PubMed: 19571809, PubMed: 19592680, PARC, NINDS, HGDP
**Nava C , et al. (2013)**	23632794	Centre de Référence déficiences intellectuelles de causes rares, Centre Diagnostic Autisme, Pitié-Salpêtrière Hospital, the Fondation Lejeune	NA
**Prasad A , et al. (2013)**	23275889	Canadian cohort	BioServe
**Nguyen LS , et al. (2013)**	23376982		
**Krumm N , et al. (2015)**	25961944	Simons Simplex Collection (SSC)	NA
**Kanduri C , et al. (2015)**	26052927	Finish cohort	Finish cohort
**Fitzgerald T , et al (2014)**	25533962	Decipher	Scottish Family Health Study
**Gazzellone MJ , et al. (2014)**	25170348	Children Development and Behavior Research Center (CDBRC)	Ontario Population Genomics Platform (OPGP), Han Chinese HapMap project, Han Chinese by Lou et al, Han Chinese Singapore Genome Variation Project
**Kushima I , et al. (2018)**	30208311	Japanese cohort	Japanese cohort
**Leppa VM , et al. (2016)**	27569545	AGRE	AGRE

**Quintela I , et al. (2017)**	28506748	Galician cohort	NA
**Girirajan S , et al. (2012)**	22970919	US cohort	NA
**Glessner JT , et al. (2009)**	19404257	NA	ACC
**Tropeano M , et al. (2013)**	23637818	NDD cohort	WTCCC2

Supplemental Information:

Shared etiology of NDDs [*1]

Several NDD-risk CNV loci have been reported to be associated with risk of more than one NDD. For instance, both deletion or duplication carriers at 16p11.2 have been found to exhibit ADHD, ID, ASD or epilepsy with significantly greater frequency than controls[1; 2]. Alternatively, it is estimated that individuals carrying monosomy in the 22q11.2 region are ~40% more likely to develop schizophrenic-spectrum disorders in adults[3], although it has also been observed that 10%-50% of patients with this same deletion[4], also known as Velocardiofacial/DiGeorge syndrome, report autism. Other behavioral disorders observed in affected individuals include attention deficit hyperactivity disorder, mood and anxiety disorders [4-6]

Other CNV loci associated with risk for both ASD and SCZ include deletions in 3q29 and 17q12, as well as duplications in 7q11.23 or 16p13.1 , in agreement with the previously reported shared etiology for these disorders[7-9].This finding also holds true for novel regions that we have detected, most of which also overlap with known risk regions. Among the newly identified regions we found some overlapping with previously known regions, such as 21q11.2-q22.3, 22q11.21-q11.23, which include the previously mentioned 21q11.2, or 8p23.3- p23.1, overlapping with 8p23.1. Few cases have been described for this terminal region of chromosome 8, and the most frequent clinical manifestations include ID[10; 11], ADHD[12] , ASD[13], and epilepsy[14]. Likewise, the overlapping regions 1p36.33-p36.22 and 1p36.33-p36.32[15; 16], 5q35.2-q35.3, or 6p terminal deletions (6p25.3, 6p25.3-p25.1)[17; 18] have also been associated with SZC, ID and ASD.

Details about other validated NDD-risk genes [*2]

In the 3q29 CNV loci we found PAK2, a candidate gene for which a high confidence level of association with NDD risk had not yet been established. However, it had been shown that this

serine/threorine kinase is essential in the regulation of cytoskeletal dynamics, and that knockout animal models recapitulated disturbed neurological synaptic patterns seen in patients with ASD[19]. In our analysis PAK2, a validated candidate gene, is closely associated with the MAPK stress activated cascade, an essential pathway in brain function, learning and memory. In iHART, a single variant has been found deleting this gene in four different families, and for two of which, all children diagnosed with ASD had this variant (Supplemental Table 3).

For CHM3, in the 1q43-44 region, cases with autism and ID have been previously reported[20]. In our analyses we not only validated its association to NDD-risk, but also found a set of clinical signs and symptoms in patients from Decipher with a statistically significant association to this NDD-risk gene: depressed nasal bridge, upslanted palpebral fissure, short neck, downturned corners of the mouth, sparse hair, micrognathia, epicanthus and hypoplasia of corpus callosum (Supplemental Table 3).

Supplemental references:

1. Niarchou, M., Chawner, S., Doherty, J.L., Maillard, A.M., Jacquemont, S., Chung, W.K., Green-Snyder, L., Bernier, R.A., Goin-Kochel, R.P., Hanson, E., et al. (2019). Psychiatric disorders in children with 16p11.2 deletion and duplication. Transl Psychiatry 9, 8.
2. Smith, H., Lane, C., Al-Jawahiri, R., and Freeth, M. (2022). Sensory processing in 16p11.2 deletion and 16p11.2 duplication. Autism Res 15, 2081-2098.
3. Schneider, M., Debbane, M., Bassett, A.S., Chow, E.W., Fung, W.L., van den Bree, M., Owen, M., Murphy, K.C., Niarchou, M., Kates, W.R., et al. (2014). Psychiatric disorders from childhood to adulthood in 22q11.2 deletion syndrome: results from the International Consortium on Brain and Behavior in 22q11.2 Deletion Syndrome. Am J Psychiatry 171, 627-639.
4. Ousley, O., Evans, A.N., Fernandez-Carriba, S., Smearman, E.L., Rockers, K., Morrier, M.J., Evans, D.W., Coleman, K., and Cubells, J. (2017). Examining the Overlap between Autism Spectrum Disorder and 22q11.2 Deletion Syndrome. Int J Mol Sci 18.
5. Bertran, M., Tagle, F.P., and Irarrazaval, M. (2018). Psychiatric manifestations of 22q11.2 deletion syndrome: a literature review. Neurologia (Engl Ed) 33, 121-128.
6. Hoeffding, L.K., Trabjerg, B.B., Olsen, L., Mazin, W., Sparso, T., Vangkilde, A., Mortensen, P.B., Pedersen, C.B., and Werge, T. (2017). Risk of Psychiatric Disorders Among Individuals With the 22q11.2 Deletion or Duplication: A Danish Nationwide, Register-Based Study. JAMA Psychiatry 74, 282-290.
7. Gandal, M.J., Haney, J.R., Parikshak, N.N., Leppa, V., Ramaswami, G., Hartl, C., Schork, A.J., Appadurai, V., Buil, A., Werge, T.M., et al. (2019). Shared Molecular Neuropathology Across Major Psychiatric Disorders Parallels Polygenic Overlap. Focus (Am Psychiatr Publ) 17, 66-72.
8. Kushima, I., Aleksic, B., Nakatochi, M., Shimamura, T., Okada, T., Uno, Y., Morikawa, M., Ishizuka, K., Shiino, T., Kimura, H., et al. (2018). Comparative Analyses of Copy-Number Variation in Autism Spectrum Disorder and Schizophrenia Reveal Etiological Overlap and Biological Insights. Cell Rep 24, 2838-2856.
9. Wu, X., Huai, C., Shen, L., Li, M., Yang, C., Zhang, J., Chen, L., Zhu, W., Fan, L., Zhou, W., et al. (2021). Genome-wide study of copy number variation implicates multiple novel loci for schizophrenia risk in Han Chinese family trios. iScience 24, 102894.
10. Hutchinson, R., Wilson, M., and Voullaire, L. (1992). Distal 8p deletion (8p23.1----8pter): a common deletion? J Med Genet 29, 407-411.
11. Wu, Y., Ji, T., Wang, J., Xiao, J., Wang, H., Li, J., Gao, Z., Yang, Y., Cai, B., Wang, L., et al. (2010). Submicroscopic subtelomeric aberrations in Chinese patients with unexplained developmental delay/mental retardation. BMC Med Genet 11, 72.
12. Shi, S., Lin, S., Chen, B., and Zhou, Y. (2017). Isolated chromosome 8p23.2-pter deletion: Novel evidence for developmental delay, intellectual disability, microcephaly and neurobehavioral disorders. Mol Med Rep 16, 6837-6845.
13. Chien, W.H., Gau, S.S., Wu, Y.Y., Huang, Y.S., Fang, J.S., Chen, Y.J., Soong, W.T., Chiu, Y.N., and Chen, C.H. (2010). Identification and molecular characterization of two novel chromosomal deletions associated with autism. Clin Genet 78, 449-456.
14. Nucaro, A., Pisano, T., Chillotti, I., Montaldo, C., and Pruna, D. (2011). Chromosome 8p23.2-pter: a critical region for mental retardation, autism and epilepsy? Clin Genet 79, 394-395; author reply 396.
15. Guo, S., Liu, J., Li, W., Yang, Y., Lv, L., Xiao, X., Li, M., Guan, F., and Luo, X.J. (2021). Genome wide association study identifies four loci for early onset schizophrenia. Transl Psychiatry 11, 248.

16. Radio, F.C., Pang, K., Ciolfi, A., Levy, M.A., Hernandez-Garcia, A., Pedace, L., Pantaleoni, F., Liu, Z., de Boer, E., Jackson, A., et al. (2021). SPEN haploinsufficiency causes a neurodevelopmental disorder overlapping proximal 1p36 deletion syndrome with an episignature of X chromosomes in females. Am J Hum Genet 108, 502-516.
17. Bogani, D., Willoughby, C., Davies, J., Kaur, K., Mirza, G., Paudyal, A., Haines, H., McKeone, R., Cadman, M., Pieles, G., et al. (2005). Dissecting the genetic complexity of human 6p deletion syndromes by using a region-specific, phenotype-driven mouse screen. Proc Natl Acad Sci U S A 102, 12477-12482.
18. Rraku, E., Kerstjens-Frederikse, W.S., Swertz, M.A., Dijkhuizen, T., van Ravenswaaij-Arts, C.M.A., and Engwerda, A. (2023). The phenotypic spectrum of terminal and subterminal 6p deletions based on a social media-derived cohort and literature review. Orphanet J Rare Dis 18, 68.
19. Wang, Y., Zeng, C., Li, J., Zhou, Z., Ju, X., Xia, S., Li, Y., Liu, A., Teng, H., Zhang, K., et al. (2018). PAK2 Haploinsufficiency Results in Synaptic Cytoskeleton Impairment and Autism-Related Behavior. Cell Rep 24, 2029-2041.
20. Khadija, B., Rjiba, K., Dimassi, S., Dahleb, W., Kammoun, M., Hannechi, H., Miladi, N., Gouider-Khouja, N., Saad, A., and Mougou-Zerelli, S. (2022). Clinical and molecular characterization of 1q43q44 deletion and corpus callosum malformations: 2 new cases and literature review. Mol Cytogenet 15, 42.
